# Pharmacokinetics of single low dose primaquine in Ugandan and Congolese children with falciparum malaria

**DOI:** 10.1016/j.ebiom.2023.104805

**Published:** 2023-09-25

**Authors:** Mavuto Mukaka, Marie A. Onyamboko, Peter Olupot-Olupot, Pimnara Peerawaranun, Kanokon Suwannasin, Watcharee Pagornrat, Jindarat Kouhathong, Wanassanan Madmanee, Winifred Were, Cate Namayanja, Peter Onyas, Harriet Titin, Joy Baseke, Rita Muhindo, Daddy K. Kayembe, Pauline O. Ndjowo, Benjamin B. Basara, Georgette S. Bongo, Charles B. Okalebo, Grace Abongo, Sophie Uyoga, Thomas N. Williams, Chiraporn Taya, Mehul Dhorda, Arjen M. Dondorp, Naomi Waithira, Mallika Imwong, Kathryn Maitland, Caterina Fanello, Nicholas P.J. Day, Joel Tarning, Nicholas J. White, Walter R.J. Taylor

**Affiliations:** a Mahidol Oxford Tropical Medicine Research Unit (MORU), Faculty of Tropical Medicine, Mahidol University, 420/6 Rajvithi Road, Bangkok, 10400, Thailand; b Centre for Tropical Medicine and Global Health, Nuffield Department of Medicine, University of Oxford, United Kingdom; c Kinshasa School of Public Health, University of Kinshasa, Avenue Tombalbaye 68-78, Democratic Republic of Congo; d Mbale Clinical Research Institute (MCRI), P.O. Box 1966, Mbale, Uganda; e Busitema University, P.O. Box 1460, Mbale, Uganda; f KEMRI-Wellcome Trust Research Programme, Kilifi, Kenya; g Institute of Global Health Innovation, Department of Surgery and Cancer, Imperial College London, SW7 2AS, United Kingdom; h Department of Molecular Tropical Medicine and Genetics, Faculty of Tropical Medicine, Mahidol University, Bangkok, Thailand

**Keywords:** Primaquine, Transmission blocking, Age-based dosing, *Plasmodium falciparum*

## Abstract

**Background:**

There are no pharmacokinetic data of single low dose primaquine (SLDPQ) as transmission blocking in African children with acute *Plasmodium falciparum* and glucose-6-phosphate dehydrogenase deficiency (G6PDd).

**Methods:**

Primaquine pharmacokinetics of age-dosed SLDPQ (shown previously to be gametocytocidal with similar tolerability as placebo) were characterised in falciparum-infected Ugandan and Congolese children aged 6 months to 11 years, treated on admission with standard 3-day dihydroartemisinin-piperaquine or artemether-lumefantrine plus SLDPQ: 6 m–<1 y: 1.25 mg, 1–5 y: 2.5 mg, 6–9 y: 5 mg, 10–11 y: 7.5 mg. LC-MS/MS-measured plasma primaquine and carboxyprimaquine (baseline, 1, 1.5, 2, 4, 8, 12, 24 h) were analysed by noncompartmental analysis. Multivariable linear regression modelled associations between covariates, including cytochrome-P450 2D6 metaboliser status, and outcomes.

**Findings:**

258 children (median age 5 [interquartile range (IQR) 3–7]) were sampled; 8 (3.1%) with early vomiting were excluded. Primaquine doses of 0.10–0.40 (median 0.21, IQR 0.16–0.25) mg base/kg resulted in primaquine maximum plasma concentrations (*C*max) of 2.3–447 (median 103.0, IQR 72.1–140.0) ng/mL between 1.0 and 8.0 (median 2) hours (T_max_) and median areas under the drug concentration curves (AUC_0-last_) 730.2 (6 m–<1 y, n = 12), 582.8 (1–5 y, n = 126), 871.1 (6–9 y, n = 80), and 931.0 (10–11 y, n = 32) ng∗h/mL. Median elimination half-live (T½) was 4.7 (IQR 3.8–5.6) hours. Primaquine clearance/kg peaked at 18 months, plateauing at 4 y. Increasing CYP2D6 metaboliser activity score [poor (3/250), intermediate (52/250), normal (150/250), ultrarapid (5/250), indeterminate (40/250)] and baseline haemoglobin were significantly associated with a lower primaquine AUC_0-last_,which increased with increasing mg/kg dose and age but was independent of the artemisinin treatment used.

**Interpretation:**

Age-dosed SLDPQ resulted in variable primaquine exposure that depended on bodyweight-adjusted dose, age, baseline haemoglobin and CYP2D6 metaboliser status, but not on dihydroartemisinin-piperaquine or artemether-lumefantrine. These data support age-dosed SLDPQ for transmission blocking in sub-Saharan Africa.

**Funding:**

This work was cofunded by the 10.13039/501100000265UK Medical Research Council, 10.13039/100010269Wellcome Trust, and UK Aid through the Global Health Trials (grant reference MR/P006973/1). The funders had no role in the study design, execution, and analysis and decisions regarding publication.

**Research in context**

**Evidence before this study**

We searched PubMed from database inception to March 1st, 2023, with no language restrictions, for studies assessing the transmission-blocking effect and pharmacokinetics of primaquine using the search terms ([Primaquine] AND [Malaria] OR [Pharmacokinetics]), and ([Primaquine] AND [Gametocytocidal] OR [Gametocytes] OR [Transmission]), and [Cytochrome 2D6].

The pharmacokinetics of primaquine has been well characterised in adults but little work has been conducted in children in general and there are no data in children acutely sick with *Plasmodium falciparum*. The best study to date was in 40 Burkinabe children aged 2–14 y with asymptomatic *P. falciparum* infection who were treated with single low dose primaquine (SLDPQ), 0.25 and 0.4 mg base/kg body weight dosed on Day 2, and artemether lumefantrine. This study reported lower primaquine and carboxyprimaquine maximal concentrations (C_max_) and exposures in younger vs. older children, consistent with age related changes in bioavailability. Independent factors for higher C_max_ and exposures were also associated with poor and intermediate vs. normal and ultrarapid, cytochrome P450 2D6, metaboliser status and increasing weight.

**Added value of this study**

This large, 250 patient pharmacokinetic study, consisting of young Ugandan and Congolese children aged 6 months to 11 years, analysed rich pharmacokinetic data by noncompartmental analysis. SLDPQ was dosed on Day 0, as recommended by the WHO, with either artemether lumefantrine or dihydroartemisinin piperaquine. These data are a subset from the safety trial of 1137 children who received aged-dosed SLDPQ/placebo in which we showed SLDPQ was similarly tolerated as placebo and was significantly gametocytocidal.

Our main findings included the wide interindividual variation in primaquine and carboxyprimaquine *C*max and exposures which was larger when dosed by age compared to weight. Primaquine clearance/kg peaked at 18 months and declined thereafter, consistent with the disposition of other drugs in children. The independent significant factors that affected the primaquine and carboxyprimaquine *C*max and exposures, in decreasing order of magnitude, were mg/kg dose of primaquine, age and baseline haemoglobin. The genotypically determined cytochrome P450 2D6 metaboliser status was only significant for primaquine exposure; a higher activity score, indicating increasing primaquine metabolism was associated with decreased primaquine exposure and there was a trend for a decreased primaquine C_max_. We observed higher primaquine and carboxyprimaquine *C*max and exposures compared to those reported in the 40 asymptomatic 2–14 y old children. These findings suggest a disease effect but other differences between the studies such as design, pharmacogenetics of the different populations, and laboratory techniques for drug concentration determination could also play a role.

**Implications of all the available evidence**

Our study adds substantial pharmacokinetic data in a hitherto unstudied population of children at high risk of contracting *P. falciparum*. They provide strong pharmacokinetic support for the age-based dosing regimen of SLDPQ and, together with its good safety profile, will increase confidence for malaria control programmes if they wish to deploy this regimen. Dosing by age is clearly associated with wider inter individual variation in primaquine disposition. By identifying the key factors responsible for this variation, we plan also to design weight-based SLDPQ regimens to match the different weight dosing bands of the commonly used artemisinin-based combination treatments.

## Introduction

Primaquine has broad antimalarial activity and plays a key role in malaria elimination. It is effective for causal prophylaxis,^1^ is gametocytocidal in *P. falciparum* infections,^2^ and provides radical cure to prevent relapses of *Plasmodium vivax* and *Plasmodium ovale* infections.^3^^,^^4^ Primaquine is metabolised mostly to an inactive metabolite, carboxyprimaquine, by the monoamine oxidase-A pathway but, importantly, several active and unstable oxidative metabolites are generated by oxidases, notably cytochrome P450 2D6.^5^ These reactive intermediates are responsible for primaquine's gametocytocidal and antirelapse activities^6^ and its key haemolytic toxicity, namely, dose-dependent, acute haemolysis in individuals with glucose-6-phosphate dehydrogenase deficiency (G6PDd).^7^

In 2012, the World Health Organization (WHO) recommended the addition of single low dose primaquine (SLDPQ) to artemisinin-based combination treatments (ACTs) to block falciparum malaria transmission from humans to vector mosquitoes. This was partly in response to the increasing spread of artemisinin-resistant *Plasmodium falciparum* (ARPf) from its epicentre in Cambodia into the Greater Mekong Subregion. The recommended target dose was 0.25 mg/kg body weight, a dose considered safe in patients with G6PDd. It replaced the earlier recommendation of adding 0.75 mg/kg of primaquine to falciparum malaria treatments. This policy was not adopted in sub-Saharan Africa and was adopted variably elsewhere.

Supporting the WHO recommendation are several studies showing that SLDPQ is well tolerated in patients with African^8^^,^^9^ and southeast Asian^10^ G6PD variants.^11^ Dose-dependent transmission blocking efficacy and reductions in gametocyte carriage have also been demonstrated with Dicko et al. suggesting a transmission blocking effect in individuals aged ≥5 years at doses ≥0.125 mg/kg of primaquine.^12^^,^^13^

There is only one pharmacokinetic study of primaquine reported in young African children. Goncalves et al. gave 0.25 and 0.4 mg/kg of primaquine on Day 2 to asymptomatic, *P. falciparum*-infected children aged 2–14 years who were treated with artemether-lumefantrine (AL).^14^ They demonstrated lower primaquine exposures in younger children given the same mg/kg dose as older children and their model predicted that, following 0.25 mg/kg of primaquine, the maximum concentration (*C*max) of primaquine in a 2-year old child (12 kg, ∼30 ng/mL) would be less than half that of a 14-year old child (40 kg, ∼73 ng/mL). Interindividual variation in the modelled *C*max was substantial: ∼7 and 16-fold for the 0.25 and 0.4 mg/kg doses, respectively. This age-dependent increase in primaquine exposures results principally from the lower drug clearance/kg seen older children and adults compared to younger children.^15^

Given the paucity of PK data in young African children, we set out to characterise primaquine and carboxyprimaquine pharmacokinetics in a subset of Ugandan and Congolese children with acute *P. falciparum* malaria who were enrolled in a large safety study of an aged-based SLDPQ regimen that demonstrated tolerability similar to placebo and gametocytocidal efficacy.^16^^,^^17^

## Methods

### Study design and participants

This was a randomised (1:1), double blind, placebo-controlled safety trial of SLDPQ combined with either open AL or dihydroartemisinin piperaquine (DHAPP). The study took place between July 2017 and November 2019 in eastern Uganda at the Mbale Regional Referral Hospital, and the Kinshasa Mahidol Oxford Research unit, on the outskirts of Kinshasa, in the Democratic Republic of Congo (DRC).

### Study conduct

We enrolled children if they were ≥6 months and <12 years and presented with acute (≤72 h), uncomplicated falciparum malaria and either a positive malaria slide for *P*. *falciparum* (mono or mixed infection) of any parasitaemia or a positive rapid diagnostic test (SD-Bioline Malaria-Ag-Pf/Pan™, SD Bioline, S. Korea), and their legal guardian gave signed informed consent to the main study and the pharmacokinetic substudy.

Using tablet strengths of 2.5, 5, 7.5 mg tablets (Centurion Laboratories, Vadodara, India)], the age-based regimen was dosed as: (i) 6 m–<1 y: 1.25 mg, (ii) 1–5 y: 2.5 mg, (iii) 6–9 y: 5 mg, and 10–11 y: 7.5 mg. AL (Coartem®, Novartis, Switzerland) and DHAPP (D-ARTEPP®, Guilin, China) were dosed by weight, according to WHO recommendations, crushed, dissolved in water and given with milk. Children aged ≥5 years in Mbale were given whole primaquine tablets to swallow. Full and half doses of all study drugs were given again, if vomiting occurred within 30 and 60 min, respectively.

There was no formal sample size calculation for this PK component of the main study; rather this depended on the capacity at each site and we aimed for a PK population of at least 200.

### Sampling and bioanalysis

Blood for drug concentrations was taken via an intravenous catheter at Day (D) 0 Hour (H) 0 and then at 1, 1.5, 2, 4, 8, 12, and 24 h. EDTA samples were centrifuged (1500–2000 × *g* for 10 min) and the plasma stored at −80 °C before analysis at the Department of Clinical Pharmacology, Mahidol-Oxford Tropical Medicine Research Unit, Bangkok, Thailand.

Primaquine and carboxyprimaquine were quantified using solid-phase extraction and high-performance liquid chromatography with mass spectrometry detection.^18^ The limits of quantification were 1.14 ng/mL and 4.88 ng/mL for plasma primaquine and carboxyPQ, respectively. Three replicates of quality control samples at low, medium, and high concentrations were analysed within each batch of clinical samples to ensure precision and accuracy during drug measurements. Total precision (i.e., relative standard deviation [SD]) for all drug measurements was <10% during drug quantification. Additional methodological details are in the Supplementary material.

### Cytochrome 2D6 molecular analysis and activity score

Fifty μL of packed red cells were collected in EDTA tubes and DNA extraction carried out using the QIAamp DNA Mini Kit (Qiagen, Germany), in accordance with the manufacturer's instructions. The molecular analysis involved three additional steps. To determine the presence of CYP2D6 gene duplications, multiplications, and deletions, long-length PCR was performed using extra-long range polymerase chain reaction, following previously published protocols (primer and probe sequences are shown in Table S1).^19^^,^^20^ Secondly, to distinguish between functional and non-functional CYP2D6, CYP2D8, and CYP2D7 genes, intron 2 sequencing was performed and, finally, to identify any mutations in the nine exons of the CYP2D6 gene, full length CYP2D6 sequence was amplified using the method of Dorado et al.^20^ From the *CYP2D6* genotyping result, the *CYP2D6* activity score was obtained to characterise the metaboliser status as poor, intermediate, normal (rapid) and ultrarapid (Table S2).^21^

### Statistical analysis

All patients who received at least one dose of ACT and SLDPQ and did not have early vomiting and had at least one quantifiable plasma primaquine concentration were included in these analyses (complete data analysis). For some analyses, mg base/kg dosing bands were grouped as: (i) 0.1–<0.15, (ii) 0.15–<0.2, (iii) 0.2–<0.25, (iv) 0.25–<0.3, and (v) 0.3–0.4. We also calculated the *C*max, AUC_0-last_ and AUC_0-∞_ in children of all ages given 0.25 (>0.24 and <0.26) mg/kg, the WHO target dose.

All analyses were done in Stata v17 (Stata Corp., Texas, USA). Proportional data between groups were analysed by chi squared or Fisher's exact test, as appropriate, and continuous data by the unpaired ‘*t*’ test, ANOVA (normally distributed data) or their nonparametric equivalents (skewed data). The correlation between two continuous variables was assessed using the Pearson or Spearman rho correlation coefficient.

Multivariable linear regression models were used to identify the factors that were independently associated with the *C*max and AUC_0-last_ outcomes using a two-step multivariable approach. The first multivariable model assessed the following independent variables: age, sex, mg/kg dose, ACT given, genotypic G6PD status (normal deficient vs. deficient/heterozygous), malaria slide vs. RDT diagnosis, CYP 2D6-defined metaboliser status, baseline temperature, haemoglobin (Hb) baseline concentration, and baseline asexual parasite count (only in those with a positive slide in a separate model, replacing slide/RDT diagnosis). Only variables with a p value < 0.05 were evaluated in the second multivariable model. The effect of CL/F and Vd/F on the age-related changes in *C*max and AUC_0-last_ were explored in the second multivariable model by adding in interaction terms of age∗CL/F (CL/F model) and age∗Vd/F (Vd/F model).

A noncompartmental approach was used to analyse the individual concentration–time data. The maximum drug concentration (*C*max) and time to maximum drug concentration (*T*max) were taken directly from the observed data. The area under the drug concentration time curve time (drug exposure) up to the last measured drug concentration (AUC_0-last_) was calculated using the cubic spline method for ascending concentrations and the logarithmic cubic spline method for descending concentrations. The terminal elimination rate constant (ke) was estimated by the log-linear best-fit regression of the observed concentrations in the terminal elimination phase. Drug exposure was extrapolated from the last observed concentration to time infinity by *C*last/ke for each subject to compute the total drug exposure (AUC_0-∞_). The terminal elimination half-life (t1/2) was estimated by Ln2/ke. The apparent volume of distribution (*Vz*/*F*) and oral clearance (CL/*F*) were calculated according to Equations (1) and (2), respectively. *Vz*/*F* is also reported as *Vz*/*F* normalised to a 70 kg individual.

For the calculation of the carboxyPQ clearance and volume of distribution, we assumed complete *in vivo* conversion of primaquine into carboxyPQ and calculated the equivalent carboxyPQ dose using the primaquine (259.347 g/mol) and carboxyPQ (274.32 g/mol) molecular weights: primaquine dose∗274.32/259.347.

(1) $$\frac{\text{CL}}{F}=\frac{\text{Dose}}{\text{AUC}0-\infty }$$

(2) $$\frac{V}{F}=\frac{CL*\frac{t1}{2}}{\mathrm{Ln}\;2}$$

### Ethics

The study protocol was approved by the: Mbale Regional Hospital Institutional Review Committee (MRHIRC), National Drug Authority, Uganda National Council for Science and Technology (UNCST), Ministry of Higher and University Education, DRC, University of Kinshasa Public Health School Ethics Committee, DRC City of Kinshasa Provincial Government, and the Oxford University Tropical Ethics Committee. The trial registration reference is ISRCTN11594437. Full details are published elsewhere.^17^ All parents/guardians gave written informed consent.

### Funding

This work was cofunded by the UK Medical Research Council, Wellcome, and UK Aid through the Global Health Trials (grant reference MR/P006973/1). The funders had no role in the study design, execution, and analysis and decisions regarding publication.

## Results

Of the 1137 children enrolled, 258 were studied but 8 (3.1%) were excluded for early vomiting (Table S3), leaving 149 children from Uganda and 101 from DRC. Baseline characteristics are detailed in Table 1. The median age was 5.5 years and 34 (13.6%) children were <2 years. Only one (0.4%) and four (1.6%) had severe and moderate malnutrition, respectively. A total of 47/149 (31.5%) Ugandan children were RDT positive for malaria but Giemsa-slide negative. Normal metaboliser status predominated over intermediate status and there were small numbers of poor or ultrarapid metabolisers; their activity scores are in Table S4. Metaboliser status could not be determined in 40 (16%) patients.

**Table 1:** Baseline characteristics of enrolled children.

Characteristics^a^	6 m–<1 y (N = 12)	1–5 y (N = 126)	6–9 y (N = 80)	10–<12 y (N = 32)	Overall (N = 250)
Age years	0.7 (0.5–0.9)	4 (1–5)	7 (6–9)	10 (10–11)	5 (0.5–11)
Sex: female n (%)	6 (50)	50 (40)	29 (36)	14 (44)	99 (40)
Weight kg	7.8 (6.6–8.8)	14.4 (7.4–24.8)	22.1 (15–36)	31.5 (18.5–42)	17.5 (6.6–42)
Dose of primaquine mg	1.25	2.5	5.0	7.5	1.25–7.5
mg/kg dose (base)	0.16 (0.14–0.19)	0.17 (0.10–0.34)	0.23 (0.14–0.33)	0.24 (0.18–0.41)	0.21 (0.10–0.41)
DHAPP n (%)	8 (75)	64 (51)	39 (49)	15 (47)	126 (50)
*Physical signs*					
MUAC cm	13.3 (11.0–15.3)	15.1 (12.0–18.0)	16.5 (12.2–21.7)	18.3 (15.5–22.1)	15.7 (11.0–22.1)
Normal nutritional status: moderate: severe malnutrition	10:1:1	124:2:0	79:1:0	32:0:0	245:4:1
Temperature °C	37.2 (35.9–39.0)	37.2 (35.9–39.9)	36.9 (36.0–39.9)	37.0 (36.2–40.0)	37.1 (35.9–40.0)
Febrile (core temp ≥38 °*C*) n (%)	4 (33)	36 (29)	13 (16)	8 (25)	61 (24)
Hepatomegaly n (%)	0 (0)	2 (2)	3 (4)	2 (6)	7 (3)
Splenomegaly n (%)	3 (25)	24 (19)	16 (20)	10 (31)	53 (21)
*Haematological parameters*					
Haemoglobin g/dL	9.3 (6.3–11.4)	10.6 (6.0–14.8)	11 (6.5–14.7)	11.6 (7.5–13.9)	10.8 (6–14.8)
Moderate anaemia (Hb <8 g/dL) n (%)	2 (17)	12 (9.5)	7 (9)	1 (3)	22 (9)
*Genotypic G6PD status* n/N (%)					
Normal male	6/12 (50)	53/126 (42)	35/80 (44)	12/31 (39)	106/249 (43)
Normal female	5/12 (42)	37/126 (29)	21/80 (26)	7/31 (23)	70/249 (28)
Hemizygous male	0/12 (0)	23/126 (18)	16/80 (20)	6/31 (19)	45/249 (18)
Homozygous female	0/12 (0)	2/126 (2)	5/80 (6)	1/31 (3)	8/249 (3)
Heterozygous females	1/12 (8)	11/126 (9)	3/80 (4)	5/31 (16)	20/249 (8)
*Cytochrome P450 2D6 status* n/N (%)					
Poor metaboliser	0/12 (0)	2/126 (2)	1/80 (1)	0/32 (0)	3/250 (1)
Intermediate metaboliser	2/12 (17)	25/126 (20)	15/80 (19)	10/32 (31)	52/250 (21)
Normal metaboliser	8/12 (67)	76/126 (60)	49/80 (61)	17/32 (53)	150/250 (60)
Ultrarapid metaboliser	1/12 (8)	3/126 (2)	1/80 (1)	0/32 (0)	5/250 (2)
Indeterminate	1/12 (8)	20/126 (16)	14/80 (18)	5/32 (16)	40/250 (16)
*Parasite data*					
Malaria RDT positive & slide negative n (%)	5 (42)	31 (25)	7 (9)	4 (12.5)	47 (19)
*Pf* asexual parasitaemia/μL Geometric mean (GSD; range)	19,405 (15; 103–241,973)	12,266 (22; 9–668,770)	11,601 (11; 65–652,806)	17,845 (11; 73–268,156)	12,862 (15; 9–668,770)
Gametocytaemic (any species) n (%)	3 (25)	25 (20)	17 (21)	4 (12.5)	49 (20)
Pf Gametocytaemia/µL Geometric mean (GSD, range)	77 (3; 27–152)	36 (3; 9–454)	60 (5; 9–2245)	56 (8; 8–1051)	47 (4; 8–2245)

MUAC—mid upper arm circumference.

RDT—rapid diagnostic test.

DHAPP—dihydroartemisinin piperaquine.

AL—artemether lumefantrine.

GSD—geometric mean.

a Continuous data are reported as median (range) unless otherwise stated.

**Fig. 1:**
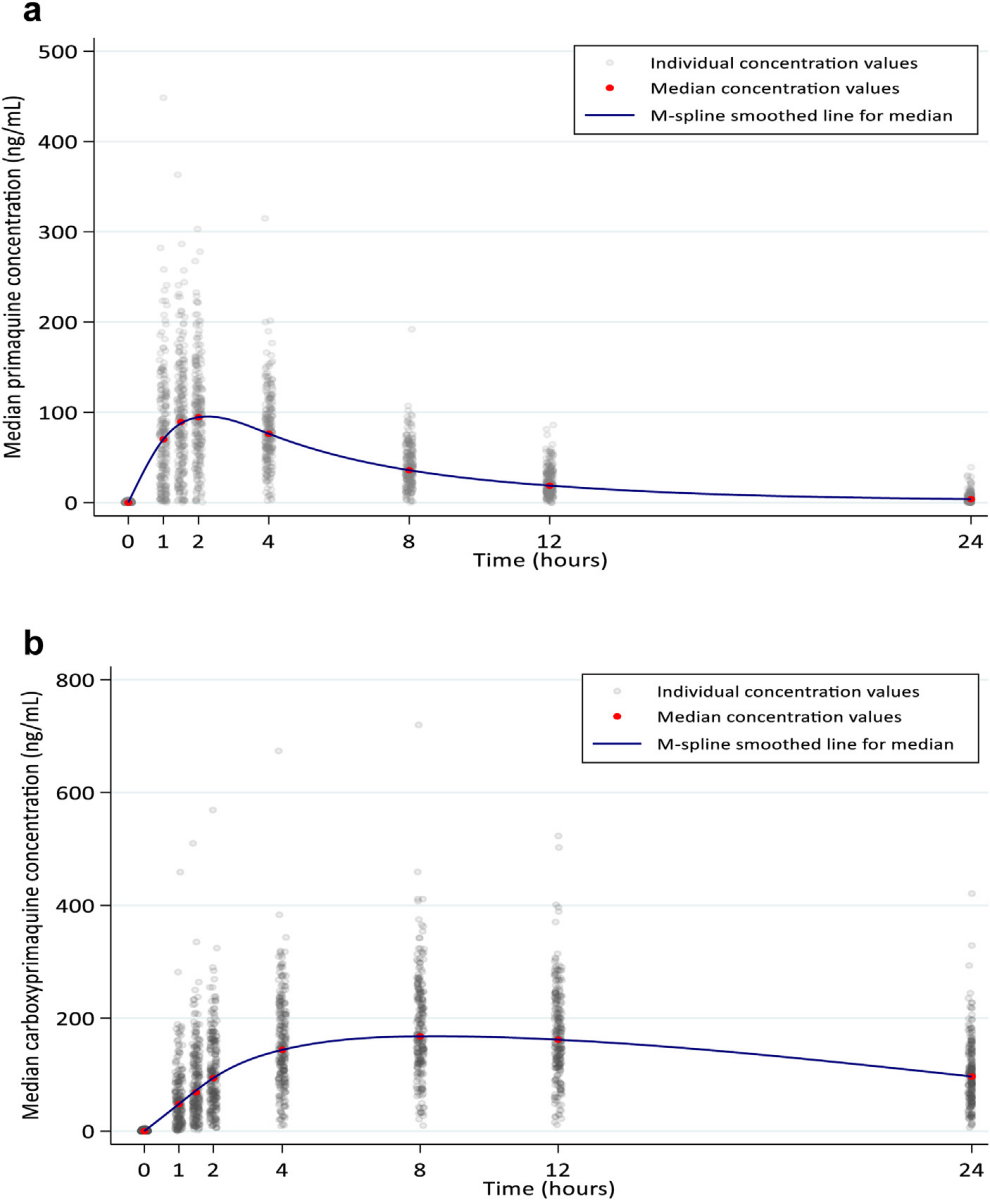
**Disposition of primaquine and of carboxyprimaquine**. a. Disposition of primaquine for all patients (n = 250). b. Disposition of carboxyprimaquine (n = 248).

For all patients combined, the median mg base/kg dose administered was 0.21, IQR 0.17–0.25, range 0.10–0.41. Primaquine was absorbed rapidly reaching a median *C*max of 103 ng/mL at a median of 2 h (Fig. 1a). The disposition of primaquine was similar in the AL and DHAPP recipients (Figure S1).

**Fig. 2:**
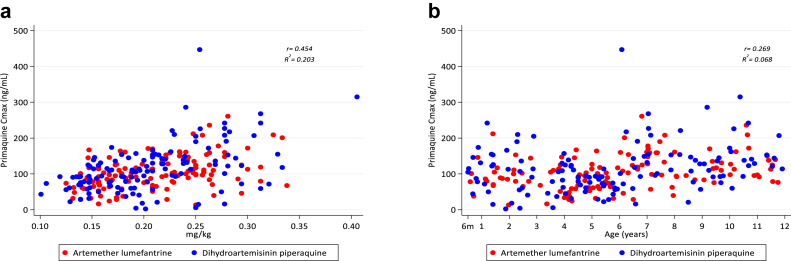
Maximum primaquine concentrations in the dihydroartemisinin piperaquine and artemether lumefantrine recipients as a function of the calculated mg/kg dose of primaquine and age.

The primaquine *C*max showed marked interindividual variation with overlap between the two ACTs (Fig. 2). Using the 95th and 5th *C*max centiles, the *C*max interindividual variation ranged from 2.7 to 27.4 for the age dosing bands and 4.1–16.2 ng/mL for the mg/kg dosing bands (Table S5). At 0.25 mg/kg (n = 29), the median *C*max was 117 (IQR 100–190, range 5.74–447) ng/mL. Primaquine PK parameters by centiles are shown in Table S6.

Median AUC_0-last_ and AUC_0-∞_ for primaquine ranged from 583.0 to 931.0 and 589.0–954.0 ng∗h/mL (Table 2), respectively. The interindividual variation in AUC_0-last_ by age was ∼3–29 fold with children aged 1, 2 and 3 years having the highest fold (23–29) in variation; by mg/kg dose, variation was ∼5–7, except for those receiving 0.25–<0.3 mg/kg, ∼26 fold (Table S5). At 0.25 mg/kg (n = 29 children of all ages), the median AUC_0-last_ and AUC_0-∞_ were 980 and 997 ng∗h/mL.

**Table 2:** Primaquine and Carboxyprimaquine pharmacokinetic parameters by dosing age group.

Primaquine	6 m–<1 y	1–5 y	6–9 y	10–<12 y	Overall
	N = 12	N = 126	N = 80	N = 32	N = 250
*C*max ng/mL	103 (78–139; 38–174)	84 (59–114; 2–242)	127 (94–159; 16–447)	134 (105–162; 60–315)	103 (72–140; 2–447)
Tmax h	2 (1–2; 1–4)	2 (2–4; 1–4)	2 (2–2; 1–4)	2 (2–2; 1–4)	2 (2–2; 1–4)
CL/F L/h	1.7 (1.2–2.5; 1.0–4.2)	4.2 (3.1–6.2; 1.5–99.6)	6.2 (4.3–8.0; 2.4–90.9)	7.9 (5.9–9.2; 3.1–12.6)	5.1 (3.3–7.7; 1–99.6)
V/F L	12.2 (9.5–17.3; 7.3–38.1)	29.0 (21.9–43.1; 9.4–1828.7)	39.3 (29.3–53.5; 14.3–1255.0)	45.7 (39.3–62.8; 23.6–84.5)	32.7 (24.3–50.3; 7.3–1828.7)
t1/2 h	4.9 (4.5–5.6; 4.3–7.1)	4.8 (4.0–5.8; 2.4–75.4)	4.5 (3.6–5.1; 2.4–19.3)	4.7 (3.8–5.6; 2.7–6.8)	4.7 (3.8–5.6; 2.4–75.4)
AUC_0-last_ ng∗h/mL	730 (514–1048; 297–1251)	583 (385–812; 15–1698)	871 (633–1202; 30–2007)	931 (767–1268; 378–2365)	718 (476–1014; 15–2365)
AUC_0-∞_ ng∗h/mL	735 (525–1057; 297–1258)	589 (403–815; 25–1703)	875 (629–1208; 55–2088)	954 (770–1276; 596–2394)	725 (483–1026; 25–2393)
Carboxyprimaquine	N = 12	N = 125^a^	N = 80	N = 31^a^	N = 248^a^
*C*max ng/mL	159 (125–214; 42–253)	139 (112–168; 11–411)	235 (198–275; 65–716)	258 (188–310; 127–350)	176 (129–242; 11–716)
Tmax h	8 (8–12; 8–12)	8 (8–12; 4–24)	8 (8–8; 4–12)	8 (8–8; 4–12)	8 (8–12; 4–24)
CL/F L/h	0.3 (0.3–0.5; 0.2–1.1)	0.8 (0.5–1.2; 0.1–8.4)	1.0 (0.7–1.3; 0.3–3.7)	1.3 (1.1–1.8; 0.5–2.5)	0.9 (0.6–1.3; 0.1–8.4)
V/F L	9.4 (6.4–12.7; 4.0–39.2)	21.2 (16.3–29.1; 6.7–279.7)	26.3 (20.7–31.7; 10.7–100.1)	33.6 (30.4–43.0; 19.4–91.5)	24.0 (18.4–31.9; 4.0–279.7)
t1/2 h	19.8 (13.7–22.1; 10.5–42.2)	17.0 (12.5–24.5; 4.5–180.2)	17.4 (12.8–24.0; 9.2–105.7)	17.4 (13.0–21.6; 10.5–62.8)	17.4 (12.8–23.9; 4.5–180.2)
AUC_0-last_ ng∗h/mL	3158 (2285–3754; 783–4920)	2426 (1669–3248; 226–7669)	4266 (3454–5157; 1149–7962)	4523 (3409–5581; 2502–6204)	3251 (2358–4445; 226–7962)
AUC_0-∞_ ng∗h/mL	4010 (2925–4964; 1151–6702)	3406 (2197–4993; 317–22,742)	5339 (4183–7435; 1428–19,723)	5709 (4120–6950; 3233–15,772)	4414 (2936–6331; 317–22,742)

Data are shown as median (IQR; range).

*C*max–maximum observed whole-blood concentration after oral administration; Tmax-observed time to reach *C*max; CL-elimination clearance; V- apparent volume of distribution; t1/2- terminal elimination half-life; AUC_0-last_- observed area under the whole-blood concentration–time curve from zero time to last observed concentration; AUC_0-∞_- predicted area under the whole-blood concentration–time curve after the last dose from zero time to infinity.

a Two participants had only one time point data over time (1 participant in 1–5 y and 1 participant in 10–11 y).

In the final multivariable model (Table 3), mg/kg dose, age and baseline Hb were consistent independent, explanatory variables for the *C*max and AUC_0-last_ values with the baseline Hb having opposite effects for primaquine and carboxyprimaquine. The effects these variables on primaquine are illustrated in Figure S2. The activity score was only significant for primaquine AUC_0-last_, trended towards significance for primaquine *C*max and carboxyprimaquine AUC_0-last_, and was not significant for the carboxyprimaquine *C*max. All examined factors in the first multivariable model are shown in Table S7.

**Table 3:** Significant independent factors in regression models to explain the maximum concentrations (*C*_max_) and exposures (AUC_0-last_) of primaquine and carboxyprimaquine in 210 *P. falciparum*-infected children.

	Slope (95% CI)	p value
Primaquine		
***C*max** (ng/mL)		
mg/kg of primaquine (base)^a^	44 (32, 56)	<0.001
Age (years)	5 (2, 7)	<0.001
D0 haemoglobin (g/dL)	−6 (−10, −2)	0.004
**AUC**_**0-last**_ (ng∗h/mL) of primaquine		
mg/kg of primaquine (base)^a^	305 (214, 397)	<0.001
Age (years)	22 (5, 39)	0.011
D0 haemoglobin (g/dL)	−30 (−59, −1)	0.040
Activity score	−166 (−258, −73)	0.001
**Carboxyprimaquine:**		
***C*max** (ng/mL)		
mg/kg of primaquine (base)^a^	82 (66, 98)	<0.001
Age (years)	7 (4, 10)	<0.001
D0 haemoglobin (g/dL)	8 (3, 13)	0.002
**AUC**_**0-last**_ (ng∗h/mL)		
mg/kg of primaquine (base)^a^	1412 (1128,1697)	<0.001
Age (years)	117 (64,170)	<0.001
D0 haemoglobin (g/dL)	150 (57,242)	0.002

These factors explained 26% and 25% of the *C*_max_ and AUC_0-last_, respectively, for primaquine and 41% and 40% of the carboxyprimaquine *C*_max_ and AUC_0-last_ variation, respectively.

For the primaquine *C*_max_, the model predicts that an increase in the dose of primaquine of 0.1 mg/kg will increase the *C*_max_ by a mean of 44 ng/mL and that an increase in age of one year will increase the *C*_max_ by a mean of 5 ng/mL. A mean decrease in *C*_max_ of 6 ng/mL is predicted to occur for every 1 g/dL increase in baseline haemoglobin e.g. a baseline Hb of 7 g/dL will reduce the *C*_max_ by a mean of 42 ng/mL whilst a 12 g/dL baseline will reduce the *C*_max_ by a mean of 72 ng/mL. An increase in the activity score of 1 is predicted to decrease the primaquine AUC_0-last_ by a mean of 166 ng^∗^h/mL.

a 0.1 mg/kg base increments.

For the primaquine *C*max, an increase in 0.1 mg/kg would result in a mean increase of 44 ng/mL whilst an increase in 1 g/dL of Hb concentration would be associated with a mean reduction of 6 ng/mL. Moreover, the model predicts a Cmax of 107.4 ng/mL and 142.2 ng/mL in a one (Hb of 10.1 g/dL) year old and 10-year-old girl or boy (Hb 11.8 g/dL), respectively, given 0.25 mg/kg of SLDPQ with AL or DHAPP and irrespective of G6PD and metaboliser status (Table 4). Corresponding data for the AUC_0-last_ also show an increase in the predicted AUC_0-last_ for a 10 y old child over a 1 y old with the intermediate metaboliser status being associated with an increased primaquine AUC_0-last_ of ∼20% (Table 4).

**Table 4:** Predicted primaquine maximum concentrations (*C*max) and exposure (AUC_0-last_) in a one and 10 year old child of any G6PD status and either CYP 2D6 intermediate metaboliser (activity score 1) or normal metaboliser status (activity score 2) given 0.25 mg base/kg of primaquine and either artemether lumefantrine or dihydroartemisinin piperaquine.

Cmax	PQ	cPQ	ratio cPQ:PQ
Age 1	107.40	186.80	1.74
Age 10	142.20	263.40	1.85
Ratio ages 1:10	0.76	0.71	–
Fold increase ages 10:1	1.32	1.41	–
AUC_0-last_	Intermediate (I) metabolisers		
Age 1	905.50	3374.00	3.73
Age 10	1052.50	4682.00	4.45
Ratio ages 1:10	0.86	0.72	–
Fold increase ages 10:1	1.16	1.39	–
AUC_0-last_	Normal (N) metabolisers		
Age 1	739.50	3374.00	4.56
Age 10	886.50	4682.00	5.28
ratio ages 1:10	0.83	0.72	–
Fold increase ages 10:1	1.20	1.39	–
AUC_0-last_ ratios & fold increases			
Ratio I:N metabolisers age 1	0.82	–	–
Fold increase I:N metabolisers age 1	1.22	–	–
Ratio I:N metabolisers age 10	0.84	–	–
Fold increase I:N metabolisers age 10	1.19	–	–

The model assumes the haemoglobin concentration is 10.1 g/dL and 11.8 g/dL in the 1 and 10 y old child, respectively; these median values were taken from the study data.

The model of Goncalves et al. predicted a *C*max of ∼ 30 ng/mL in a 2-year old, 12 kg child and ∼73 ng/mL in a 14-year old, 40 kg child (41%) when dosed with 0.25 mg/kg of primaquine. Corresponding data from our model (assuming it is valid for a 14 y old) are 112.4 and 162.2 ng/mL (69.3%).

With increasing age, the reductions in primaquine *C*max and AUC_0-last_ associated with CL/F were small for a given mg/kg dose of primaquine. There was no significant CL/F-age interaction for the carboxyprimaquine *C*max and AUC_0-last_. The effect of Vd/F on age related changes in *C*max and AUC_0-last_ was variable, being associated with a small decrease in primaquine *C*max and a trend to a small decrease in primaquine AUC_0-last_. The effect on the carboxyprimaquine *C*max and AUC_0-last_ were very small increases in both parameters with increasing age for a given primaquine mg/kg dose (Tables S8 & S9).

**Fig. 3:**
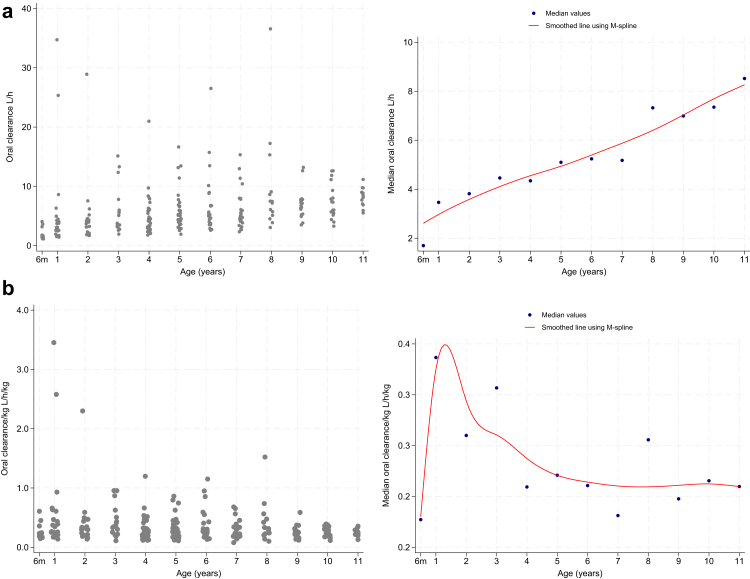
Median oral clearance of primaquine in L/h as a function of age, from 6 months to 11 years, and adjusted for body weight. Spearman rho correlation coefficients were 0.97 (p < 0.0001) and −0.37 (p < 0.0001). a. Clearance b. Clearance adjusted for body weight.

Primaquine half-lives ranged from 1 to 8 h for medians of 4.5–4.9 h across the age bands (Table 2). The median apparent oral clearance increased with increasing age but the weight-adjusted median values rose to peak at ∼18 months of age and declined thereafter (Fig. 3). Vd/F increased with increasing weight (Figure S3) and, when normalised to a 70 kg individual, showed a decreasing trend with increasing weight (Figure S4); similar but less distinct trends were seen for age (data not shown).

Primaquine was metabolised to carboxyprimaquine which reached a median *C*max of 175.5 ng/mL at a median *T*max of 8 h (Table 2). The disposition of carboxyprimaquine for all patients is shown in Fig. 1b & Figure S5. The median half-life, clearance, and Vd/F were 17.4 h, 0.93 L/h, and 24.0 L, respectively. The carboxyprimaquine PK parameters by age are shown in Table 2 and by centiles in Table S6. Factors independently associated with a higher carboxyprimaquine *C*max and AUC_0-last_ were increasing mg/kg dose, age and a higher baseline Hb (Table 3).

For all patients combined, median carboxyprimaquine to primaquine ratios were: (i) 1.7: 1 for *C*max (175.5 vs. 103 ng/mL), (ii) 4.5: 1 for AUC_0-last_ (718.5 vs. 3250.7 ng∗h/mL), and (iii) 6.1: 1 for AUC_0-∞_ (4413.8 vs. 725.4 ng∗h/mL). These ratios are very similar to those predicted by our model (Table 4).

## Discussion

In this large prospective study, we characterised the pharmacokinetic properties of primaquine and carboxyprimaquine in children with acute falciparum malaria given a newly-proposed, age-based regimen of SLDPQ that was well tolerated and showed gametocytocidal efficacy.^17^

There was wide interindividual variation in maximum plasma concentrations and exposures of primaquine and carboxyprimaquine, notably in the younger children. Key explanatory factors in addition to the weight-adjusted dose were activity score, age and baseline haemoglobin concentrations with the latter deceasing primaquine but increasing the carboxyprimaquine *C*max and AUC_0-last_. Primaquine is bioactivated by oxidases, notable the polymorphic CYP 2D6. The activity of this enzyme inferred from the genotype was significantly associated with primaquine exposure.

We observed increasing weight-adjusted primaquine clearance that peaked in children aged ∼18 months, then declined to plateau at 4 y (Fig. 3; the peak CL/F/kg was ∼60% higher than the plateau. High drug clearance is common in young children and our observations are consistent with allometric theory, which predicts an increase in CL/F/kg from birth to peak at 2 y, followed by a gradual curvilinear decline to reach adult values.^15^ The primaquine Vd/F increased with increasing age and weight but the weight adjusted Vd/F was higher in lighter children consistent with their greater proportions of total body water and fat compared with older children.^22^

There was wider interindividual variation in primaquine *C*max when dosed by age compared to weight of ∼3–27 fold (∼9 fold difference) vs. ∼4–16 fold (∼4 fold difference), respectively, consistent with 7–16 fold interindividual variation reported by Goncalves et al. in weight-dosed, asymptomatic *P. falciparum*-infected, 2 to 14 y old children^14^ and the 8–22 (∼3) fold difference reported in 12 well and 12 malnourished, vivax-infected Indian children, respectively, of mean age 10 y, given 0.3 mg/kg/day.^23^ Thus, weight-based dosing roughly reduced the overall *C*max variability by ∼50% (4/9) in our children. The mg/kg dose was the most significant covariate in determining the *C*max and AUC_0-last_ and was ∼10 fold greater compared to age for both parameters.

We found a significant independent and inverse effect of CYP2D6 metaboliser status on primaquine exposure and a trend for primaquine *C*max and carboxyprimaquine exposure. Goncalves et al. found that a CYP2D6 adjusted CL/F resulted in a better model fit and a clear relationship between higher primaquine and carboxyprimaquine exposures and lower CYP2D6 activity scores.^14^ They also estimated that children aged a little over 4 y would have half the bioavailability (*F*_50_) of a mature person, supporting an age dependent increase in bioavailability as contributing to higher exposures with age.

When dosed at 0.25 mg/kg, the measured median primaquine in children of all ages was *C*max was 117 ng/mL, similar to the 115 ng/mL modelled from healthy Papuan children aged 5–12 y given 0.5 mg/kg of primaquine without an ACT^24^ but ∼2-fold higher than the 50 ng/mL modelled by Goncalves et al. in children given 0.25 mg/kg on D2.^14^ At this dose, our model predicts a 1 y old child would have a *C*max ∼75% (107 ng/mL/142 ng/mL) of that of a 10 y old child, and almost 70% for a 2 vs. 14 y old child. This is a larger difference than that predicted by Goncalves et al. (41%) in the 2 and 14 y old children.^14^ Similarly, our measured median primaquine AUC_0-∞_ of 997 h∗ng/mL was double the 450 h∗ng/mL predicted by Goncalves et al. and modelled exposure in a 1 y old is >80% of that in a 10 y old (for both intermediate and normal metabolisers). The median carboxyPQ:PQ *C*max ratio, 1.7:1, was lower than ∼6:1 reported in healthy adults^25^, ^26^, ^27^ but very similar to the ∼1.5:1 observed in adults with falciparum malaria treated with quinine.^28^ Our carboxyPQ:PQ exposure ratios (4–5:1) were also considerably less than the ∼17–25:1 reported in healthy adults.^25^, ^26^, ^27^ Taken together, our data suggest a disease effect increasing primaquine exposure and reducing metabolism to carboxyprimaquine but we cannot exclude differences in study designs and possible pharmacogenetic differences with other populations.

Acute *P. falciparum* malaria increases the acute phase protein alpha-1-acid glycoprotein, the main primaquine plasma binding protein,^29^ resulting in greater retention of primaquine in plasma and a concomitant reduction in tissue distribution (i.e., Vd/F).^30^ Increased primaquine *C*max and reduced Vd/F also result from interactions with DHAPP(26), chloroquine,^27^ artesunate pyronaridine^25^ and, very likely, by AL, given the similar AL and DHAPP *C*max values in our study. Reduced primaquine clearance resulting from malaria related hepatic dysfunction^31^ has been documented in falciparum malaria^31^ and would explain the low carboxyprimaquine to primaquine ratios seen in malaria.^28^ We found an independent association of a higher primaquine *C*max and exposure and lower carboxyprimaquine values with lower baseline haemoglobin concentrations but no association with the D0 parasitaemia or baseline fever. The aetiology of anaemia in malaria is multifactorial so this could be a chance finding but, if reconfirmed by others, it might suggest that a lower haemoglobin concentration is a crude marker of disease severity in uncomplicated malaria.

Our study had several limitations. Some patients were malaria slide negative but RDT positive, suggesting a resolving infection but this did not significantly affect the primaquine *C*max or AUC_0-last_ in the multivariable analyses. Moreover, patients were only recruited if they had a clear history of fever or a measured fever at presentation. Non malaria fever may also affect PQ pharmacokinetics in a similar way to malaria. The number of malnourished children was very small so we could not assess primaquine disposition in this vulnerable group. This is an area requiring further study to add to the paucity of literature.^23^ Although we identified a substantial number of intermediate and normal primaquine metabolisers, there were too few children who were poor or ultrarapid metabolisers to assess their effect on *C*max and AUC_0-last_ but the overall assessment of activity scores indicated increased exposures with lower scores. We did not measure any CYP2D6 mediated hydroxylated metabolites of primaquine (e.g. 5-hydroxyprimaquine and 5,6 orthoquinone) which are key to PQs transmission blocking efficacy^6^ and haemolytic toxicity in G6PD deficient individuals.^32^ 5,6-orthoquinone can be measured in red blood cells,^7^ urine^33^ and, recently, Birrel et al. have measured the oxidative metabolites of tafenoquine in blood, plasma and urine.^34^ These measures may define PK PD relationships better and inform SLDPQ optimal dosing.

### Conclusions

This large pharmacokinetic study of primaquine and carboxyprimaquine in *P. falciparum*-infected children, treated with DHAPP or AL, demonstrated wide interindividual variation in *C*max and AUC_0-last_, and the age effects on *C*max and oral clearance. Primaquine Cmax and exposure were higher compared to earlier work in children with asymptomatic falciparum infection. These results provide the PK foundation for the good tolerability and gametocytocidal efficacy of age-dosed SLDPQ and support the suitability of this regimen for transmission blocking in sub-Saharan Africa.

## Contributors

WRJT conceived the study and co-wrote the grant with NPJD. WRJT, NPJD, AD, MAO, CF, PO-O, TNW, KM, MD and JT developed the protocol. PO-O, MAO, WW, CN, PO, HT, JB, BB, GB, RM, DK, PON, CBO, GA, SU, TNW, CF, and KM were responsible for data collection. Site PIs were PO-O (Mbale) and MAO (KIMORU). MAO, CF, PO-O, TNW and KM oversaw the study. KS, JK, WM, MI performed the CYP2D6 genotyping. WRJT, MM, PP, CT analysed the data and wrote the first draft of the paper with significant intellectual input from NJW and JT. All authors have seen and approved the final submitted version and agreed to publication. WRJT, MM, and NW verified the data.

## Data sharing statement

Selected data generated and analysed during this study are included in this published article and its supplementary information files. Deidentified individual participant data will be available after publication to applicants who provide a sound proposal to the Mahidol Oxford Tropical Medicine Research Unit Data Access Committee. They can contact the corresponding author in the first instance.

## Declaration of interests

All authors declare that they have no competing interests.

## Appendix A Supplementary data

Supplemental Tables

BAKMAL1606_PAC_Protocol
