# Supplementary material for: Pharmacokinetics of single low dose primaquine in Ugandan and Congolese children with falciparum malaria
Source: eBioMedicine. 2023 Sep 25;96:104805. doi: 10.1016/j.ebiom.2023.104805 (PMC10550634; doi:10.1016/j.ebiom.2023.104805)
Supplement: Supplemental Tables [file mmc1.docx]

# Table S1. Primer and probe sequences for detecting CYP 2D6 mutations after Dorado and Puapraset.

| Analysis | | Primer and probe names | Sequences (5’-3’) | Length(bp) | GC  (%) | T_m_  (^o^C) | Amplicon size (bp) |
| --- | --- | --- | --- | --- | --- | --- | --- |
| **XL-PCR**  ^2^ | | DPKup  DPKlow | 5′-GTTATCCCAGAAGGCTTTGCAGGCTTCA-3′  5′-GCCGACTGAGCCCTGGGAGGTAGGTA-3′ | 28  26 | 50.0  65.4 | 67.8  71.1 | 5100 |
|  |  | 2D6dupl-F  2D6dupl-R | 5′-CCTGGGAAGGCCCCATGGAAG-3′  5′-CAGTTACGGCAGTGGTCAGCT-3′ | 21  21 | 66.7  57.1 | 65.5  63.2 | 3500 |
|  |  | 5′2D6*5  3′2D6*5 | 5′-CACCAGGCACCTGTACTCCTC-3′  5′-CAGGCATGAGCTAAGGCACCCAGAC-3′ | 21  25 | 61.9  60.0 | 62.7  67.9 | 3500 |
| **Int2** | | 5′2D6Int2  3′2D6Int2 | 5′-TTTTGCACTGTGGGTCCTC-3′  5′-CAAGGTGGACACGGAGAAG-3′ | 19  19 | 52.6  57.9 | 58.5  58.4 | 1,101 |
| Direct sequencing of PCR-reamplified products  **DSP** | | 5′2D6Ex1 3′2D6Ex1 | 5′-GCACAGTCAACACAGCAGGT-3′  5′-AATGCCCTTCTCCAGGAAGT-3′ | 20 20 | 55.050.0 | 61.759.2 | 503 |
|  |  | 5′2D6Ex2  3′2D6Ex2 | 5′-TTCCTCCATCACAGAAGGTG-3′  5′-CTCCCTAGTGCAGGTGGTTT-3′ | 20  20 | 50.0  55.0 | 57.4  59.9 | 501 |
|  |  | 5′2D6Ex34  3′2D6Ex34 | 5′-GTCTTCCCTGAGTGCAAAGG-3′  5′-AGTGGGGTCTCCTGGAATG-3′ | 20  19 | 55.0  57.9 | 59.1  58.9 | 754 |
|  |  | 5′2D6Ex56  3′2D6Ex56 | 5′-GAGGGACTTGGTGAGGTCAG-3′  5′-GACACTCCTTCTTGCCTCCT-3′ | 20  20 | 60.0  55.0 | 60.0  59.6 | 794 |
|  |  | 5′2D6Ex7  3′2D6Ex7 | 5′-ATGAACTTTGCTGGGACACC-3′  5′-CCAGCCCTGCCTATACTCTG-3′ | 20  20 | 50.0  60.0 | 59.0  59.9 | 505 |
|  |  | 5′2D6Ex89  3′2D6Ex89 | 5′-TCTAGTGGGGAGACAAACCAG-3′  5′-CTGAGGAGGATGATCCCAAC-3′ | 21  20 | 52.4  55.0 | 59.3  57.7 | 802 |
| Allele-specific oligonucleotide probes real time SNPs genotyping, **ASO** | C100T | 5′2D6C100T  3′2D6C100T  2D6C100T_WT  2D6C100T_MT | 5′-CCTGGTGGACCTGATGCA-3′  5′-CCCGGGCAGTGGCA -3′  5′-CCTGGTG**G**GTAGCGTG-3′  5′-CCTGGTG**A**GTAGCGTG-3′ | 18  14  16  16 | 61.1  78.6  69.0  63.0 | 59.5  58.7  51.1  48.5 | 73 |
|  | G1846A | 5′2D6G1846A  3′2D6G1846A  2D6G1846A_WT  2D6G1846A_MT | 5′-GACCCCTTACCCGCATCTC-3′  5′-GCTCACGGCTTTGTCCAAGA-3′  5′-CCCCCA**G**GACGCC-3′  5′-CCCCCA**A**GACGCC-3′ | 19  20  13  13 | 63.2  55.0  85.0  77.0 | 60.1  61.5  48.0  46.0 | 73 |
|  | C2850T | 5′2D6C2850T  3′2D6 C2850T  2D6C2850T _WT  2D6C2850T _MT | 5′-CCTGAGAGCAGCTTCAATGATGA-3′  5′-CCATCCCGGCAGAGAACAG-3′  5′-ACTATGC**G**CAGGTTC -3′  5′-CACTATGC**A**CAGGTTC-3′ | 23  19  15  16 | 47.8  63.2  53.0  50.0 | 61.3  60.7  41.9  43.4 | 67 |
|  | G4180C | 5′2D6G4180C  3′2D6G4180C  2D6G4180C _WT  2D6G4180C _MT | 5′-CCACCATGGTGTCTTTGCTTTC-3′  5′-GCACAGCACAAAGCTCATAGG-3′  5′-CTGGTGA**G**CCCATCC-3′  5′-CTGGTGA**C**CCCATCC-3′ | 22  21  15  15 | 50.0  52.4  67.0  67.0 | 60.9  60.4  47.4  47.4 | 67 |

Dorado P, Caceres MC, Pozo-Guisado E, Wong ML, Licinio J, Llerena A. Development of a PCR-based strategy for CYP2D6 genotyping including gene multiplication of worldwide potential use. *Biotechniques* 2005; **39**(4): 571-4.

Puaprasert K, Chu C, Saralamba N, et al. Real time PCR detection of common CYP2D6 genetic variants and its application in a Karen population study. *Malar J* 2018; **17**(1): 427.

# Table S2. Cytochrome 2D6 status and activity score.

| CYP2D6 phenotype | Activity Score |
| --- | --- |
| CYP2D6 Ultrarapid Metaboliser | > 2.5 |
| CYP2D6 Normal Metaboliser | 1.25 - 2.25 |
| CYP2D6 Intermediate Metaboliser | 0.25* - 1 |
| CYP2D6 Poor Metaboliser | 0 |
| Indeterminate | n/a |

* the CYP2D6*10 score was reduced to 0.25 in March 2019 ([Final-Consensus-CYP2D6-genotype-to-phenotype-table_-final_Mar2019.pdf (cpicpgx.org)](https://cpicpgx.org/wp-content/uploads/2019/03/Final-Consensus-CYP2D6-genotype-to-phenotype-table_-final_Mar2019.pdf)) ^1^

1. Caudle KE, Sangkuhl K, Whirl-Carrillo M, et al. Standardizing CYP2D6 Genotype to Phenotype Translation: Consensus Recommendations from the Clinical Pharmacogenetics Implementation Consortium and Dutch Pharmacogenetics Working Group. *Clin Transl Sci* 2020; **13**(1): 116-24.

# Table S3. Characteristics of the eight children who had early vomiting.

| Age (years) | Sex | Length of illness (days) | History of vomiting | Day 0 temperature ^0^C | Parasitaemia  N/µL | Primaquine dose mg | ACT given |
| --- | --- | --- | --- | --- | --- | --- | --- |
| 0.8 | Female | 3 | No | 36.7 | negative | 1.25 | AL |
| 0.9 | Male | 0 | No | 38.8 | negative | 1.25 | DHAPP |
| 1 | Male | 0 | Yes | 38.1 | 264,453.3 | 1.25 | AL |
| 2 | Female | 2 | Yes | 37 | negative | 2.5 | AL |
| 5 | Male | 0 | No | 38.6 | 337,437 | 2.5 | AL |
| 7 | Male | 2 | Yes | 38.3 | 186,516 | 5 | DHAPP |
| 7 | Male | 2 | No | 38.2 | 57,148 | 5 | DHAPP |
| 7 | Male | 1 | Yes | 38.8 | 180.6 | 5 | DHAPP |

# Table S4. Metaboliser activity scores in 250 children.

| Activity score | Number | Percent | Cumulative percent |
| --- | --- | --- | --- |
| 0 | 3 | 1.2 | 1.2 |
| 0.25 | 1 | 0.4 | 1.6 |
| 0.5 | 13 | 5.2 | 6.8 |
| 0.75 | 4 | 1.6 | 8.4 |
| 1 | 34 | 13.6 | 22 |
| 1.5 | 85 | 34 | 56 |
| 2 | 65 | 26 | 82 |
| 3 | 5 | 2 | 84 |
| Unknown | 40 | 16 | 100 |

# Table S5. Centile breakdown of the maximum concentrations of primaquine in ng/mL and the AUC_0-last_ in ng*h/mL as a function of mg/kg administered dose and in individual ages from 6 months to 11 years. The fold differences are based on the 95^th^ and 5^th^ centiles and are ranked in ascending order.

| Primaquine C*_max_* | | | | | | | | | |
| --- | --- | --- | --- | --- | --- | --- | --- | --- | --- |
| N* | mg/kg band | min | p5 | p25 | p50 | p75 | p95 | max | fold |
| 72 | 0.2-<0.25 | 2.3 | 51 | 93.6 | 124.5 | 148 | 210 | 286 | 4.1 |
| 38 | 0.1-<0.15 | 22.4 | 28.5 | 55.9 | 73.6 | 92.2 | 145 | 167 | 5.1 |
| 15 | 0.3-0.4 | 59.2 | 59.2 | 79.6 | 155 | 209 | 315 | 315 | 5.3 |
| 77 | 0.15-<0.2 | 4.28 | 24.2 | 62.4 | 91.8 | 114 | 157 | 174 | 6.5 |
| 48 | 0.25-<0.3 | 5.74 | 14.9 | 97 | 125 | 190 | 242 | 447 | 16.2 |
| N* | age | min | p5 | p25 | p50 | p75 | p95 | max | fold |
| 14 | 11 | 76.3 | 76.3 | 97.2 | 123 | 144 | 207 | 207 | 2.7 |
| 18 | 9 | 75.5 | 75.5 | 92.5 | 116 | 136 | 286 | 286 | 3.8 |
| 24 | 7 | 39.7 | 57.1 | 99.9 | 150.5 | 174.5 | 227 | 268 | 4.0 |
| 33 | 5 | 22.4 | 28.5 | 58.3 | 73.8 | 92.3 | 127 | 164 | 4.5 |
| 12 | 0.5 | 37.9 | 37.9 | 78.1 | 103 | 138.5 | 174 | 174 | 4.6 |
| 33 | 4 | 24.2 | 28.4 | 52.9 | 79.1 | 103 | 145 | 167 | 5.1 |
| 18 | 10 | 59.9 | 59.9 | 113 | 139.5 | 209 | 315 | 315 | 5.3 |
| 24 | 6 | 16 | 28.7 | 96.85 | 121.5 | 160.5 | 261 | 447 | 9.1 |
| 14 | 8 | 20.9 | 20.9 | 83.5 | 101.7 | 147 | 221 | 221 | 10.6 |
| 22 | 1 | 2.3 | 13.3 | 70.3 | 88.25 | 126 | 212 | 242 | 15.9 |
| 20 | 2 | 4.28 | 11.24 | 62.8 | 101 | 140 | 207.5 | 210 | 18.5 |
| 18 | 3 | 5.74 | 5.74 | 51 | 93.75 | 114 | 157 | 157 | 27.4 |
| Primaquine AUC_0-last_ | | | | | | | | | |
| N* | mg/kg band | min | p5 | p25 | p50 | p75 | p95 | max | fold |
| 72 | 0.2-<0.25 | 14.6 | 291.6 | 633.2 | 802.8 | 1095.5 | 1488.6 | 1836.9 | 5.1 |
| 15 | 0.3-0.4 | 372.9 | 372.9 | 574.6 | 1024.0 | 1628.0 | 2364.7 | 2364.7 | 6.3 |
| 77 | 0.15-<0.2 | 28.4 | 186.4 | 424.7 | 671.0 | 880.8 | 1211.6 | 1668.7 | 6.5 |
| 38 | 0.1-<0.15 | 111.1 | 149.9 | 323.6 | 475.3 | 619.9 | 1107.3 | 1469.6 | 7.4 |
| 48 | 0.25-<0.3 | 29.8 | 69.3 | 589.9 | 941.8 | 1294.1 | 1783.6 | 1999.6 | 25.7 |
| number | age | min | p5 | p25 | p50 | p75 | p95 | max | fold |
| 14 | 11 | 377.5 | 377.5 | 770.1 | 859.3 | 1065.1 | 1296.3 | 1296.3 | 3.4 |
| 18 | 9 | 381.7 | 381.7 | 656.1 | 741.6 | 1024.3 | 1446.0 | 1446.0 | 3.8 |
| 18 | 10 | 569.8 | 569.8 | 673.1 | 1019.0 | 1358.1 | 2364.7 | 2364.7 | 4.2 |
| 12 | 0.5 | 296.5 | 296.5 | 513.6 | 730.2 | 1047.9 | 1251.0 | 1251.0 | 4.2 |
| 24 | 7 | 327.5 | 380.9 | 635.6 | 985.2 | 1251.1 | 1783.6 | 2006.6 | 4.7 |
| 33 | 5 | 149.9 | 183.9 | 359.3 | 486.2 | 625.0 | 958.0 | 1219.0 | 5.2 |
| 33 | 4 | 111.1 | 225.2 | 402.0 | 567.4 | 837.8 | 1189.6 | 1469.6 | 5.3 |
| 24 | 6 | 29.8 | 185.3 | 555.0 | 952.9 | 1238.4 | 1789.9 | 1999.6 | 9.7 |
| 14 | 8 | 127.1 | 127.1 | 549.1 | 680.4 | 1006.0 | 1637.1 | 1637.1 | 12.9 |
| 22 | 1 | 14.6 | 69.3 | 497.5 | 718.5 | 1056.1 | 1603.5 | 1697.8 | 23.1 |
| 20 | 2 | 28.4 | 56.2 | 510.8 | 647.4 | 886.2 | 1404.1 | 1464.9 | 25.0 |
| 18 | 3 | 38.8 | 38.8 | 309.5 | 574.0 | 812.0 | 1137.1 | 1137.1 | 29.3 |

N* - number of patents

# Table S6. Pharmacokinetic parameters for primaquine and carboxyprimaquine in each age dosing band broken down by centiles.

|  | Minimum | p1 | p5 | p25 | p50 | p75 | p95 | p99 | Maximum |
| --- | --- | --- | --- | --- | --- | --- | --- | --- | --- |
| Primaquine |  |  |  |  |  |  |  |  |  |
| 6m-<1y (N=12) |  |  |  |  |  |  |  |  |  |
| Cmax ng/mL | 37.9 | 37.9 | 37.9 | 78.1 | 103.0 | 138.5 | 174.0 | 174.0 | 174.0 |
| Tmax h | 1.0 | 1.0 | 1.0 | 1.3 | 2.0 | 2.0 | 4.0 | 4.0 | 4.0 |
| CL/F L/h | 1.0 | 1.0 | 1.0 | 1.2 | 1.7 | 2.5 | 4.2 | 4.2 | 4.2 |
| V/F L | 7.3 | 7.3 | 7.3 | 9.5 | 12.2 | 17.3 | 38.1 | 38.1 | 38.1 |
| t1/2 h | 4.3 | 4.3 | 4.3 | 4.5 | 4.9 | 5.6 | 7.1 | 7.1 | 7.1 |
| AUC_0-last_ ng*h/mL | 296.5 | 296.5 | 296.5 | 513.6 | 730.2 | 1,047.9 | 1,251.0 | 1,251.0 | 1,251.0 |
| AUC_0-∞_ ng*h/mL | 297.3 | 297.3 | 297.3 | 524.5 | 734.8 | 1,056.9 | 1,257.7 | 1,257.7 | 1,257.7 |
| 1-5y (N=126) |  |  |  |  |  |  |  |  |  |
| Cmax ng/mL | 2.3 | 4.3 | 18.2 | 58.5 | 83.7 | 114.0 | 166.0 | 212.0 | 242.0 |
| Tmax h | 1.0 | 1.0 | 1.0 | 1.5 | 2.0 | 4.0 | 4.0 | 4.0 | 4.0 |
| CL/F L/h | 1.5 | 1.5 | 1.9 | 3.1 | 4.2 | 6.2 | 21.3 | 84.3 | 99.6 |
| V/F L | 9.4 | 10.8 | 15.4 | 21.9 | 29.0 | 43.1 | 145.2 | 658.4 | 1,828.7 |
| t1/2 h | 2.4 | 2.4 | 3.1 | 4.0 | 4.8 | 5.8 | 7.6 | 23.2 | 75.4 |
| AUC_0-last_ ng*h/mL | 14.6 | 28.4 | 111.1 | 385.3 | 582.8 | 812.0 | 1,343.3 | 1,603.5 | 1,697.8 |
| AUC_0-∞_ ng*h/mL | 25.1 | 29.7 | 117.6 | 402.6 | 588.8 | 814.9 | 1,344.1 | 1,646.1 | 1,702.7 |
| 6-9y (N=80) |  |  |  |  |  |  |  |  |  |
| Cmax ng/mL | 16.0 | 16.0 | 42.8 | 94.3 | 126.5 | 159.0 | 244.0 | 447.0 | 447.0 |
| Tmax h | 1.0 | 1.0 | 1.0 | 1.5 | 2.0 | 2.0 | 4.0 | 4.0 | 4.0 |
| CL/F L/h | 2.4 | 2.4 | 2.7 | 4.3 | 6.2 | 8.0 | 17.1 | 90.9 | 90.9 |
| V/F L | 14.3 | 14.3 | 16.3 | 29.3 | 39.3 | 53.5 | 110.9 | 1,255.0 | 1,255.0 |
| t1/2 h | 2.4 | 2.4 | 2.7 | 3.6 | 4.5 | 5.1 | 7.7 | 19.3 | 19.3 |
| AUC_0-last_ ng*h/mL | 29.8 | 29.8 | 306.7 | 633.2 | 871.1 | 1,201.7 | 1,726.2 | 2,006.6 | 2,006.6 |
| AUC_0-∞_ ng*h/mL | 55.0 | 55.0 | 291.9 | 628.5 | 875.2 | 1,208.3 | 1,843.2 | 2,087.8 | 2,087.8 |
| 10y to 11y (N=32) |  |  |  |  |  |  |  |  |  |
| Cmax ng/mL | 59.9 | 59.9 | 76.3 | 105.3 | 133.5 | 162.0 | 242.0 | 315.0 | 315.0 |
| Tmax h | 1.0 | 1.0 | 1.0 | 1.5 | 1.8 | 2.0 | 4.0 | 4.0 | 4.0 |
| CL/F L/h | 3.1 | 3.1 | 4.0 | 5.9 | 7.9 | 9.2 | 12.4 | 12.6 | 12.6 |
| V/F L | 23.6 | 23.6 | 28.1 | 39.3 | 45.7 | 62.8 | 79.1 | 84.5 | 84.5 |
| t1/2 h | 2.7 | 2.7 | 3.0 | 3.8 | 4.7 | 5.6 | 6.5 | 6.8 | 6.8 |
| AUC_0-last_ ng*h/mL | 377.5 | 377.5 | 569.8 | 766.7 | 931.0 | 1,267.6 | 1,836.9 | 2,364.7 | 2,364.7 |
| AUC_0-∞_ ng*h/mL | 595.7 | 595.7 | 603.9 | 769.9 | 954.4 | 1,275.7 | 1,859.8 | 2,393.5 | 2,393.5 |
| Carboxyprimaquine |  |  |  |  |  |  |  |  |  |
| 6m-<1y (N=12) |  |  |  |  |  |  |  |  |  |
| Cmax ng/mL | 42.4 | 42.4 | 42.4 | 125.0 | 158.5 | 213.5 | 253.0 | 253.0 | 253.0 |
| Tmax h | 8.0 | 8.0 | 8.0 | 8.0 | 8.0 | 12.0 | 12.0 | 12.0 | 12.0 |
| CL/F L/h | 0.2 | 0.2 | 0.2 | 0.3 | 0.3 | 0.5 | 1.1 | 1.1 | 1.1 |
| V/F L | 4.0 | 4.0 | 4.0 | 6.4 | 9.4 | 12.7 | 39.2 | 39.2 | 39.2 |
| t1/2 h | 10.5 | 10.5 | 10.5 | 13.7 | 19.8 | 22.1 | 42.2 | 42.2 | 42.2 |
| AUC_0-last_ ng*h/mL | 782.5 | 782.5 | 782.5 | 2,284.8 | 3,157.7 | 3,753.9 | 4,920.3 | 4,920.3 | 4,920.3 |
| AUC_0-∞_ ng*h/mL | 1,150.5 | 1,150.5 | 1,150.5 | 2,925.0 | 4,010.2 | 4,963.6 | 6,701.6 | 6,701.6 | 6,701.6 |
| 1-5y (N=125) |  |  |  |  |  |  |  |  |  |
| Cmax ng/mL | 11.0 | 18.0 | 42.8 | 112.0 | 139.0 | 168.0 | 243.0 | 297.0 | 411.0 |
| Tmax h | 4.0 | 4.0 | 4.0 | 8.0 | 8.0 | 12.0 | 12.0 | 24.0 | 24.0 |
| CL/F L/h | 0.1 | 0.1 | 0.3 | 0.5 | 0.8 | 1.2 | 2.8 | 5.6 | 8.4 |
| V/F L | 6.7 | 7.2 | 9.2 | 16.3 | 21.2 | 29.1 | 60.8 | 193.6 | 279.7 |
| t1/2 h | 4.5 | 8.4 | 10.0 | 12.5 | 17.0 | 24.5 | 56.6 | 163.6 | 180.2 |
| AUC_0-last_ ng*h/mL | 225.6 | 302.8 | 807.0 | 1,669.2 | 2,426.1 | 3,248.0 | 4,899.8 | 5,713.1 | 7,668.9 |
| AUC_0-∞_ ng*h/mL | 316.6 | 472.4 | 933.1 | 2,197.0 | 3,406.0 | 4,992.5 | 9,002.9 | 18,820.5 | 22,741.6 |
| 6-9y (N=80) |  |  |  |  |  |  |  |  |  |
| Cmax ng/mL | 64.9 | 64.9 | 104.5 | 197.5 | 235.0 | 274.5 | 386.5 | 716.0 | 716.0 |
| Tmax h | 4.0 | 4.0 | 4.0 | 8.0 | 8.0 | 8.0 | 12.0 | 12.0 | 12.0 |
| CL/F L/h | 0.3 | 0.3 | 0.4 | 0.7 | 1.0 | 1.3 | 2.8 | 3.7 | 3.7 |
| V/F L | 10.7 | 10.7 | 14.6 | 20.7 | 26.3 | 31.7 | 55.9 | 100.1 | 100.1 |
| t1/2 h | 9.2 | 9.2 | 9.7 | 12.8 | 17.4 | 24.0 | 55.9 | 105.7 | 105.7 |
| AUC_0-last_ ng*h/mL | 1,148.6 | 1,148.6 | 2,010.4 | 3,454.0 | 4,265.8 | 5,156.8 | 6,876.9 | 7,962.0 | 7,962.0 |
| AUC_0-∞_ ng*h/mL | 1,428.0 | 1,428.0 | 1,895.3 | 4,182.7 | 5,339.0 | 7,434.7 | 15,412.2 | 19,723.0 | 19,723.0 |
| 10y to 11y (N=31) |  |  |  |  |  |  |  |  |  |
| Cmax ng/mL | 127.0 | 127.0 | 131.0 | 188.0 | 258.0 | 310.0 | 345.0 | 350.0 | 350.0 |
| Tmax h | 4.0 | 4.0 | 4.0 | 8.0 | 8.0 | 8.0 | 12.0 | 12.0 | 12.0 |
| CL/F L/h | 0.5 | 0.5 | 0.8 | 1.1 | 1.3 | 1.8 | 2.1 | 2.5 | 2.5 |
| V/F L | 19.4 | 19.4 | 20.7 | 30.4 | 33.6 | 43.0 | 60.9 | 91.5 | 91.5 |
| t1/2 h | 10.5 | 10.5 | 10.5 | 13.0 | 17.4 | 21.6 | 39.8 | 62.8 | 62.8 |
| AUC_0-last_ ng*h/mL | 2,501.9 | 2,501.9 | 2,580.8 | 3,409.4 | 4,522.9 | 5,580.9 | 6,025.2 | 6,204.3 | 6,204.3 |
| AUC_0-∞_ ng*h/mL | 3,233.3 | 3,233.3 | 3,718.5 | 4,119.5 | 5,709.1 | 6,950.3 | 9,919.8 | 15,772.4 | 15,772.4 |

Cmax, maximum observed whole-blood concentration after oral administration; Tmax, observed time to reach Cmax; CL, elimination clearance; V, apparent volume of distribution; t1/2, terminal elimination half-life; AUC0–last, observed area under the whole-blood concentration-time curve from zero time to last observed concentration; AUC0–∞, predicted area under the whole-blood concentration-time curve after the last dose from zero time to infinity.

# Table S7. Independent and non-significant factors associated with C*_max_* and AUC_0-last_ of primaquine and carboxyprimaquine. All factors explained 31% and 28% of the C*_max_* and AUC_0-last_ variation of primaquine, respectively and all factors explained 46% and 41% of the C*_max_* and AUC_0-last_ variation of carboxyprimaquine, respectively.

|  | Slope (95% CI) | P value |
| --- | --- | --- |
| Primaquine: |  |  |
| Full multivariable model |  |  |
| **Cmax** (ng/mL) |  |  |
| Mg/kg of primaquine* | 46 (33, 59) | <0.001 |
| Age (years) | 4 (2, 7) | 0.002 |
| D0 haemoglobin (g/dL) | -7 (-11, -3) | 0.002 |
| G6PD normal (reference) vs. deficient† | -15 (-30, 0.04) | 0.051 |
| Treatment: AL (reference) vs. DHAPP‡ | 12 (-2, 26) | 0.084 |
| Male (reference) vs. female | 2 (-12, 16) | 0.796 |
| Ln parasitaemia | 0.06 (-3, 3) | 0.965 |
| D0 temperature | -0.6 (-8, 7) | 0.871 |
| Activity score | -12 (-24, 0.8) | 0.066 |
| Full multivariable model |  |  |
| **AUC_0-last_** (ng*h/mL) of primaquine |  |  |
| Mg/kg of primaquine* | 323 (227, 419) | <0.001 |
| Age (years) | 21 (3, 40) | 0.026 |
| D0 haemoglobin (g/dL) | -41 (-72, -10) | 0.011 |
| G6PD normal (reference) vs. deficient† | -102 (-215, 12) | 0.078 |
| Treatment: AL (reference) vs. DHAPP‡ | -8 (-114, 97) | 0.879 |
| Male (reference) vs. female | 23 (-84, 130) | 0.667 |
| Ln parasitaemia | 9 (-11, 28) | 0.377 |
| D0 temperature | 48 (-10, 105) | 0.102 |
| Activity score | -153 (-246, -59) | 0.002 |
| Carboxyprimaquine: |  |  |
| Full multivariable model |  |  |
| **Cmax** (ng/mL) |  |  |
| Mg/kg of carboxyprimaquine* | 83 (66, 100) | <0.001 |
| Age (years) | 5 (2, 8) | 0.004 |
| D0 haemoglobin (g/dL) | 9 (3, 14) | 0.002 |
| G6PD normal (reference) vs. deficient† | -14 (-34, 6) | 0.176 |
| Treatment: AL (reference) vs. DHAPP‡ | -6 (-25, 13) | 0.516 |
| Male (reference) vs. female | 2 (-17, 21) | 0.862 |
| Ln parasitaemia | -2 (-5, 2) | 0.318 |
| D0 temperature | -7 (-17, 4) | 0.203 |
| Activity score | -10 (-27, 6) | 0.219 |
| Full multivariable model |  |  |
| **AUC_0-last_** (ng*h/mL) of carboxyprimaquine |  |  |
| Mg/kg of carboxyprimaquine* | 1,487 (1,152, 1821) | <0.001 |
| Age (years) | 86 (19, 152) | 0.012 |
| D0 haemoglobin (g/dL) | 177 (68, 285) | 0.002 |
| G6PD normal (reference) vs. deficient† | -187 (-582, 208) | 0.351 |
| Treatment: AL (reference) vs. DHAPP‡ | -188 (-556, 180) | 0.315 |
| Male (reference) vs. female | -31 (-405, 342) | 0.868 |
| Ln parasitaemia | -21 (-89, 47) | 0.546 |
| D0 temperature | -37 (-237, 162) | 0.712 |
| Activity score | -282 (-609, 45) | 0.091 |

* 0.1 mg/kg increments

† G6PD deficient includes hemizygous males, homo- and heterozygous females

‡ AL – artemether lumefantrine, DHAPP – dihydroartemisinin piperaquine

# Table S8. Assessment of the age effect on primaquine and carboxyprimaquine due to clearance using an interaction test in the final model. All factors explained 43% and 46% of the C*_max_* and AUC_0-last_ variation of primaquine, respectively, and 49% and 51% of the C*_max_* and AUC_0-last_ variation of carboxyprimaquine, respectively.

|  | Slope (95% CI) | P value |
| --- | --- | --- |
| Primaquine: |  |  |
| ***C*max** (ng/mL) |  |  |
| Mg/kg of primaquine* | 46 (35, 57) | <0.0001 |
| Age (years) | 7 (4, 10) | <0.0001 |
| D0 haemoglobin (g/dL) | -3 (-7, 0.005) | 0.050 |
| Clearance (CL/F L/h) | -1 (-2, -0.1) | 0.026 |
| Age x Clearance | -0.3 (-0.6, -0.1) | 0.002 |
| **AUC_0-last_** (ng*h/mL) of primaquine |  |  |
| Mg/kg of primaquine** | 320 (242, 398) | <0.0001 |
| Age (years) | 38 (19, 57) | 0.0001 |
| D0 haemoglobin (g/dL) | -13 (-38, 12) | 0.316 |
| Activity score | -129 (-208, -50) | 0.002 |
| Clearance (CL/F L/h) | -8 (-14, -2) | 0.008 |
| Age x Clearance | -2 (-4, -0.9) | 0.002 |
| Carboxyprimaquine: |  |  |
| ***C*max** (ng/mL) |  |  |
| Mg/kg of carboxyprimaquine* | 85 (70, 99) | <0.0001 |
| Age (years) | 5 (0.9, 9) | 0.016 |
| D0 haemoglobin (g/dL) | 10 (5, 15) | <0.0001 |
| Clearance (CL/F L/h) | -4 (-6, -2) | <0.0001 |
| Age x Clearance | 0.3 (-0.08, 0.7) | 0.126 |
| **AUC_0-last_** (ng*h/mL) |  |  |
| Mg/kg of carboxyprimaquine * | 1,466 (1,208, 1,723) | <0.0001 |
| Age (years) | 96 (30, 163) | 0.005 |
| D0 haemoglobin (g/dL) | 196 (112, 281) | <0.0001 |
| Clearance (CL/F L/h) | -70 (-100, -41) | <0.0001 |
| Age x Clearance | 3 (-3, 10) | 0.315 |

# Table S9. Assessment of the age effect on primaquine and carboxyprimaquine due to the volume of distribution using an interaction test in the final model. All factors explained 35% and 35% of the C*_max_* and AUC_0-last_ variation of primaquine, respectively, and 46% and 48% of the C*_max_* and AUC_0-last_ variation of carboxyprimaquine, respectively.

|  | Slope (95% CI) | P value |
| --- | --- | --- |
| Primaquine |  |  |
| ***C*max** (ng/mL) |  |  |
| Mg/kg of primaquine* | 45 (34, 57) | <0.0001 |
| Age (years) | 5 (3, 8) | <0.0001 |
| D0 haemoglobin (g/dL) | -4 (-8, -0.6) | 0.023 |
| Volume of distribution (V/F L) | -0.04 (-0.1, 0.02) | 0.180 |
| Age x Volume of distribution | -0.02 (-0.04, -0.01) | 0.006 |
| **AUC_0-last_** (ng*h/mL) of primaquine |  |  |
| Mg/kg of primaquine** | 314 (227, 400) | <0.0001 |
| Age (years) | 25 (7, 42) | 0.005 |
| D0 haemoglobin (g/dL) | -19 (-47, 8) | 0.172 |
| Activity score | -156 (-243, -69) | 0.001 |
| Volume of distribution (V/F L) | -0.4 (-0.9, 0.002) | 0.051 |
| Age x Volume of distribution | -0.1 (-0.3, -0.002) | 0.047 |
| Carboxyprimaquine |  |  |
| ***C*max** (ng/mL) |  |  |
| Mg/kg of carboxyprimaquine* | 83 (68, 99) | <0.0001 |
| Age (years) | 3 (-0.5, 7) | 0.092 |
| D0 haemoglobin (g/dL) | 9 (4, 14) | 0.0002 |
| Volume of distribution (V/F L) | -0.5 (-0.8, -0.3) | <0.0001 |
| Age x Volume of distribution | 0.07 (0.02, 0.1) | 0.003 |
| **AUC_0-last_** (ng*h/mL) |  |  |
| Mg/kg of carboxyprimaquine * | 1,445 (1,180, 1,710) | <0.0001 |
| Age (years) | 54 (-9, 117) | 0.095 |
| D0 haemoglobin (g/dL) | 181 (94, 268) | 0.0001 |
| Volume of distribution (V/F L) | -11 (-15, -6) | <0.0001 |
| Age x Volume of distribution | 1 (0.5, 2) | 0.003 |

# Methodological details of the LC-MS measurement of the plasma samples for primaquine and carboxyprimaquine concentrations.

Chemicals & reagents

Racemic primaquine (PRQ) and carboxyprimaquine (CPRQ) were provided by the WorldWide Antimalarial

Resistance Network (WWARN) and stable isotope-labelled primaquine and carboxyprimaquine were provided by Prof Larry Walker, National Centre for Natural Products Research, University of Mississippi, USA. LC–MS grade acetonitrile, methanol and water were obtained from J.T. Baker (Phillipsburg, NJ, USA). Dimethyl sulphoxide (AR grade), ammonium acetate and ammonium formate (LC–MS grade) were obtained from Fluka/Sigma-Aldrich (Sigma-

Aldrich, St. Louis, MO, USA). Formic acid (LC–MS grade) was obtained from Merck (Merck Darmstadt, Germany).

Instrumentation, separation and detection

Sample preparation and solid-phase extraction was performed on an automated Freedom Evo 200 platform

(TECAN, Mannedorf, Switzerland). The LC system was an Agilent 1200 system consisting of a binary LC pump,

a vacuum degasser, a temperature-controlled micro well plate auto-sampler set at 4 °C and a column compartment

set at 20 °C (Agilent technologies, Santa Clara, CA, USA).

The mass spectrometry equipment was an API 5000 triple quadrupole system (Applied Biosystems/MDS Sciex, Foster City, CA, USA), with a TurboV Ionization Source (TIS) interface operated in the positive ion mode. Data acquisition was performed using Analyst 1.5 (Applied Biosystems/MDS Sciex, Foster City, CA, USA). PRQ, PRQ-D3, CPRQ and CPRQ-D3 enantiomers were separated on a Chiralcel OD-3R column (150 mm × 4.6 mm, I.D. 3 μm; Chiral Technologies Inc., West Chester, PA, USA), protected by a Chiralcel OD-3R guard column (4 mm × 10 mm, I.D. 3 μm), at a flow rate of 1.0 mL/min. Mobile phase A contained 20 mM ammonium formate:acetonitrile, 75:25 v/v with 0.1% formic acid, and mobile phase B contained methanol:acetonitrile (75:25, v/v). The following gradient program was employed for separation of enantiomeric PRQ and CPRQ and to remove phospholipid residues from the column: 0–15 min (100% mobile phase A), 15–16.0 min (100% mobile phase A to 100% mobile phase B), 16.0–20.8 min (100% mobile phase B), 20.8–21 min (100% mobile phase B to 100% mobile phase A), and 21.0–26.5 min (100% mobile phase A). The MS/MS conditions were optimized by infusing PRQ (10 ng/mL) and CPRQ (20 ng/mL) at 10 μL/min, using a Harvard infusion pump connected directly to the MS. Additional MS/MS tuning was performed by continuous infusion of PRQ (25 ng/mL) and CPRQ (50 ng/mL) at a flow rate of 20 μL/min via a “T”-connector into the post-column mobile phase at a flow rate of 1.0 mL/min.

TIS temperature was maintained at 700 °C and the TIS voltage was set to 4500 V. The curtain gas was set to 30 psi and the ion source gas 1 (GS1) and ion source gas 2 (GS2) at 50 and 60 psi, respectively. The CAD gas in the collision cell was set to 5 psi. Quantification was performed using multiple reaction monitoring (MRM) for the transitions m/z 260–175 and m/z 263–86 for PRQ and PRQ-D3, respectively, and m/z 275–175 and m/z 278–178 for CPRQ and CPRQ-D3, respectively. The declustering potential (DP) was set to 60 V for all analytes and stable isotope-labelled internal standards.

Preparation of standards and quality control samples

Stock solutions of PRQ (0.5 mg/mL) and PRQD3 (1 mg/mL) were prepared in acetonitrile:water (50:50, v/v) and stock solutions of CPRQ (1 mg/mL) and CPRQ-D3 (1 mg/mL) were prepared in dimethylsulfoxide:methanol (1:3, v/v). Working solutions were prepared by serial dilutions in acetonitrile:water (50:50, v/v). Working solutions of PRQ-D3 and CPRQ-D3 (5 μg/mL and 25 μg/mL, respectively) were stored in 100 μL aliquots at − 80 °C until analysis. Calibration standards and quality control (QC) samples were prepared by adding working solution to human EDTA plasma. The total content of working solution was less or equal to 2% of the total plasma volume in all cases except for the over curve dilution sample where it was 4%. Six calibration standards, excluding zero concentration, were prepared at 0.571–260 ng/mL for each enantiomer of PRQ and 2.44–2,500 ng/mL for each enantiomer of CPRQ, and stored at − 80 °C until analysis. Quality control samples for accuracy and precision of each enantiomer of PRQ and CPRQ were prepared at 3 × lower limit of quantification (LLOQ), mid-range and upper range (i.e. 1.46, 16.8 and 195 ng/mL for PRQ, and 7.32, 117 and 1,875 ng/mL for CPRQ). All QC samples were stored at − 80 °C until analysis.

Analytical procedure

Plasma samples (100 μL) were aliquoted onto a 1 mL 96-wellplate. Solid phase extraction was performed by

adding precipitation solution (300 μL of 1% formic acid in acetonitrile) containing 5 ng/mL of PRQ-D3 and

50 ng/mL of CPRQ-D3. The 96-well plate was covered with a methanol-washed seal mat and mixed on a Mixmate (Eppendorf, Germany) at 1000 rpm for 2 min followed by centrifugation at 1100g for 5 min. 200 μL of

supernatant was loaded directly onto the phospholipid removal SPE (solid-phase extraction) plate HybrideSPEPhospholipid, Sulpelco, USA) and passed through by continuously increasing the vacuum. The eluent was

diluted with 200 μL of dilution solution, containing methanol and 20 mM ammonium formate (75:25, v/v).

The elution sample plate was covered with a methanol washed Nunc pre-slit seal mat, mixed on a Mixmate at

900 rpm for 2 min and centrifuged at 1100 g for 2 min. A total volume of 5 μL was injected into the LC–MS/MS

system.

Calibration and linearity

The calibration curve was set to 0.571–260 ng/mL and 2.44–2,500 ng/mL, for each enantiomer of PRQ and

CPRQ, respectively. These calibration curves covered the ranges of expected therapeutic drug concentrations

in plasma, based on observations following a single oral dose of 30 mg (base) primaquine phosphate in volunteers. Each calibration level was run in duplicate except at the lower limit of quantification (LLOQ), which was run in five replicates during the four days of validation. Linear and quadratic regression models of the calibration curve response (peak area ratios of PRQ/PRQ-D3 and CPRQ/CRQ-D3) with and without weighting (1/x and 1/x2) were evaluated for each calibration curve. The optimal regression model was chosen on the basis of back-calculated concentrations of calibration standards (i.e., relative bias of back-calculated concentrations compared to nominal values) as well as the accuracy of predicted QC samples.

Precision and accuracy

Precision and accuracy were assessed by daily analysis of five replicates of QC samples (three concentration levels),

LLOQ samples and upper limit of quantification (ULOQ) samples over the four days of validation. Five replicates

of over-curve dilution were assessed by a five-fold dilution of spiked standards at 1038 and 10,000 ng/mL of

PRQ and CPRQ, respectively. Intra-, inter- and total-assay precision of QC samples, LLOQ, ULOQ and

over curve dilution samples were calculated using analysis of variance (ANOVA). The acceptance criteria for accuracy and precision was + 15% except for the LLOQ, which was + 20%.

Hanpithakpong W, Day NPJ, White NJ, Tarning J. Simultaneous and enantiospecific quantification of primaquine and carboxyprimaquine in human plasma using liquid chromatography-tandem mass spectrometry. Malar J 2022; 21(1): 169.
