# Supplementary material for: Pharmacokinetics of single low dose primaquine in Ugandan and Congolese children with falciparum malaria
Source: eBioMedicine. 2023 Sep 25;96:104805. doi: 10.1016/j.ebiom.2023.104805 (PMC10550634; doi:10.1016/j.ebiom.2023.104805)
Supplement: BAKMAL1606_PAC_Protocol [file mmc2.pdf]

Assessing the tolerability and safety of single low dose primaquine in African children with acute uncomplicated falciparum malaria and glucose 6 phosphate dehydrogenase deficiency

Version 2 (01 September 20188)

Short Title: Primaquine in African Children – PAC study

Study sponsor:

The Masters and Scholars of the University of Oxford,

Oxford

United Kingdom.

OxTREC reference number: 53-16

Investigators

Principal investigator	Prof. Nicholas JP Day, MBChB, FRCP	Mahidol Oxford Tropical Medicine Clinical Research unit (MORU),<br>Mahidol University,<br>420/6 Rajvithi road, Rajthevee,<br>Bangkok,<br>10400,<br>Thailand.<br>&<br>Centre for Tropical Medicine and Global Health,<br>Nuffield Department of Medicine,<br>University of Oxford,<br>United Kingdom	Tel: 662-203-6333<br>nickd@tropmedres.ac
CoPI & Clinical Coordinator	Dr. Bob Taylor, MD, FRCP		Tel: 662-203-6333<br>bob@tropmedres.ac
Co-investigator	Dr. Caterina Fanello, PhD		Tel:662-203-6333<br>Caterina@tropmedres.ac
Co-investigator	Prof. Arjen Dondorp, MD, PhD		Tel: 662-203-6333 # 6373<br>arjen@tropmedres.ac
Study statistician	Dr. Mavuto Mukaka, PhD		Tel: 662-203-6333<br>Mavuto@tropmedres.ac
Laboratory coordinator	Dr. Mehul Dhorda, PhD		Tel: 662-203-6333<br>mehul.dhorda@wwarn.org
PK coordinator	Prof. Joel Tarning		Tel: 662-203-6333<br>joel@tropmedres.ac
Site PI Democratic Republic of Congo	Prof. Marie Onyamboko, MD	Kinshasa School of Public Health, University of Kinshasa,<br>Avenue Tombalbaye 68-78, DRC	Tel: 0990024201<br>akatshimarie@yahoo.fr
Site PI Uganda	Dr. Peter Olupot-Olupot,<br>MBChB, MPH, PhD	Mbale Clinical Research Institute,<br>Dept. of Paediatrics, Mbale Regional Referral Hospital, Pallisa Road Zone,<br>P.O Box 921. Mbale Uganda	Tel: +256 772457217<br>polupotolupot@yahoo.com
Co-investigator	Prof. Kathryn Maitland<br>MBBS, PhD, FRCP	Imperial College of Science & Technology<br>Wellcome Trust Centre for Global Health<br>Room 232, Wright Fleming Institute<br>St Mary's Campus<br>Norfolk Place, Paddington<br>London, W2 1PG	Tel: +2544733411022<br>kathryn.maitland@gmail.com
Co-investigator	Prof. Tom Williams<br>MBBS, PhD, FRCP		Tel: +254733411021<br>tom.n.williams@gmail.com

Approved by:

Signature………………………………………………… Date ……………………………..

Name ……………………………………………………

Confidentiality Statement

This document contains confidential information that must not be disclosed to anyone other than the Sponsor, the Investigator Team and members of the Research Ethics Committee. This information cannot be used for any purpose other than the evaluation or conduct of the clinical investigation without the prior written consent of the principal investigator.

ABBREVIATIONS

ACT	Artemisinin based combination treatment
AE	Adverse event
AL	Artemether lumefantrine
AS	Artesunate
CarboxyPQ	Carboxyprimaquine
CRFs	Case Report Forms
CTCAE	Common Terminology Criteria for Adverse Events
DHAPP	Dihydroartemisinin piperaquine
DMEC	Drug Monitoring and Ethics Committee
DRC	Democratic Republic of Congo
EC	Ethics Committee
G6PD	Glucose-6-Phosphate Dehydrogenase
GCP	Good Clinical Practice
HNV	Human Normal Volunteer
IBD	Inherited blood disorder
ICH	International Conference on Harmonization
KEMRI	Kenya Medical Research Institute
KIMORU	Kinshasa Mahidol Oxford Tropical Medicine Research unit
KM	Kaplan Meier
MDA	Mass Drug Administration
MoH	Ministry of Health
MORU	Mahidol Oxford Tropical Medicine Research unit
MRC UK	Medical Research Council, United Kingdom
MTA	Material Transfer Agreement
PCR	Polymerase Chain Reaction
PD	Pharmacodynamic
PI	Principal investigator
PK	Pharmacokinetic
PQ	Primaquine
SAE	Serious Adverse Event
SPC	Summary of Product Characteristics
WT	Wellcome Trust

TABLE OF CONTENTS

1. BACKGROUND & RATIONALE 9
2. AIMS & OBJECTIVES 10
2.1 Primary Objective 10
2.2 Secondary Objectives 10
3. STUDY METHODS 11
3.1 Study sites 11
3.2 Study design 11
3.3 Primary Endpoint 11
3.4 Secondary endpoints 11
3.5 Inclusion criteria 12
3.6 Exclusion Criteria 12
3.7 Initial assessment at outpatients or clinic 12
3.7.1 Uganda 13
3.7.2 Kinshasa 13
3.7.3 Screening failure / caregiver decline 14
3.8 Enrolment into the study 14
3.9 G6PD status 14
3.10 Drug allocation and randomisation 14
3.10.1 Primaquine/placebo 15
3.10.2 Primaquine/placebo administration 15
3.10.3 Balancing recruitment numbers 16
3.10.4 Option to dose primaquine/placebo on Day 1 16
3.10.5 Patients not completing treatment 16
3.10.6 Admission to hospital 17
3.10.7 Follow up 17
3.10.8 Blood volume 17
3.10.9 Duration of Study 17
4. WITHDRAWAL & LOST TO FOLLOW UP 17
4.1 PI withdrawal 17
4.2 Patient withdrawal 18
4.3 Loss to follow-up 18
4.4 Unscheduled visits 18
4.5 Protocol deviations & violations 18
5. STUDY DRUGS 19
5.1 Artemether lumefantrine 19

5.2 Dihydroartemisinin piperaquine 19
5.3 Primaquine/placebo 20
5.4 Drug supervision 21
5.5 Concomitant drugs that must not be prescribed 21
5.6 Concomitant drugs that are allowed 21
5.7 Concomitant drugs that may interfere with primaquine 22
6. RESCUE TREATMENT 23
6.1 Parenteral rescue treatment 23
6.2 Oral rescue treatment 23
7. ASSESSMENTS 23
7.1 Clinical evaluations 23
7.1.1 History, symptoms & examination 23
7.1.2 Reading malaria blood films 23
7.1.3 Gametocyte quantification by qPCR 24
7.1.4 Blood film for reticulocyte count 24
7.1.5 Full blood count, HemoCue & HCT measurements 24
7.1.6 Standard spectrophotometric quantification of G6PD enzyme activity 24
7.1.7 G6PD Biosensor 24
7.1.8 The G6PD rapid diagnostic test 24
7.1.9 G6PD genotype 25
7.1.10 Characterising sickle cell disease and alpha thalassaemia 25
7.1.11 CYP 2D6 genotype 25
7.1.12 Percutaneous measurement of methaemoglobin 25
7.1.13 Specimen labelling 25
7.2 Primaquine, lumefantrine and piperaquine concentrations 26
7.3 Sample Shipment 26
8. SAFETY MONITORING & ADVERSE EVENTS 27
8.1 Adverse events & severity 27
8.2 Relatedness of Adverse Event to study drugs 27
8.3 Adverse Event Recording 27
8.4 Patient Management of Adverse Events 28
8.5 Serious Adverse Events 28
8.6 Expedited Reporting of Serious Adverse Events 28
9. DATA MONITORING & ETHICS COMMITTEE 28
10. MANAGEMENT OF SEVERE / PROFOUND ANAEMIA 29
10.1 General principles 29
10.2 Indications for blood transfusion include: 29

11. STATISTICS 29
11.1 Sample size 29
11.2 Analyses & analysis populations 30
12. ETHICS 31
12.1 Informed Consent 31
12.2 Approval by the national ethics committees 31
12.3 Risks 32
12.3.1 Artemether lumefantrine 32
12.3.2 Dihydroartemisinin piperaquine 32
12.3.3 Primaquine 32
12.3.4 Blood sampling 33
12.4 Benefits 33
12.5 Alternatives to joining the study 33
12.6 Patient reimbursements 33
12.7 Confidentiality 34
12.8 Clinical trials insurance 34
12.9 Clinical trial registration 34
12.10 Monitoring procedures 34
13. DATA HANDLING AND RECORD KEEPING 34
14. FINANCING 35
15. DATA OWNERSHIP & PUBLICATION POLICY 35
16. REFERENCES 36
17. APPENDIX 1: STUDY SCHEDULE – KINSHASA & UGANDA INPATIENTS 40
18. APPENDIX 2: STUDY SCHEDULE – UGANDA OUTPATIENTS 41
19. APPENDIX 3. WHO CLASSIFICATION OF THERAPEUTIC EFFICACY 42

Amendment history

Amendment No.	Protocol Version No.	Date issued	Author(s) of changes
1	2.0	01 Sep 2018	Bob Taylor<br>Peter Olupot-Olupot<br>Caterina Fanello<br>Prayoon Yuentrakul
**Details of Changes made**			
• Added Joel Tarning as PK coordinator (List of investigators)<br>• Replaced ‘parent’ with ‘caregiver’ throughout the document<br>• Added option of testing for G6PD status using FST (Sections 3.2 and 3.9)<br>• Revised inclusion age from 11 to <12 years for clarity (Section 3.5)			

- Revised severe anaemia to Hb<6g/dL in the exclusion criteria (Section 3.6)
- Revised the admission to hospital to allow more flexibility during the first few days in the hospital.(Section 3.10.6)
- Hb data will be sent weekly to DMEC (Section 3.10.6)
- Revised the blood volume for clarity (Section 3.10.8)
- Revised consent withdrawal for clarity (Section 4.2)
- Revised window period for follow-up visit (Section 4.3)
- DHAPP dosing is revised to match with the DHAPP formulations available onsite (Section 5.2)
- Revised 30 mins to 60 mins for observation after drug administration (Section 5.4)
- Gametocyte quantification by qPCR technique is revised (Section 7.1.3)
- Reduced number of patients for PK sampling from 400 to 300 per site (Section 7.2)
- Added the USA where samples will be sent to (Section 7.3)
- Revised the adverse event recording to record all grades (Section 8.3)
- Revised indication for transfusion, i.e. Hb< 4-.6 is now 5 g/dL and removed hyperparasitaemia (Section 10.2)

1 BACKGROUND & RATIONALE

In September 2012, the World Health Organization (WHO) Evidence Review Group (ERG) recommended adding single low-dose primaquine (SLDPQ, 0.25 mg base/kg body weight, 15 mg for adults) to artemisinin combination therapies (ACTs), without testing for glucose-6-phosphate dehydrogenase deficiency (G6PDd), in nonpregnant, *P. falciparum* infected patients aged ≥1 year (later amended to ≥6 months) to block *Plasmodium falciparum* gametocyte transmission from humans to mosquitoes [1 2].

This recommendation was aimed primarily at countries of the Greater Mekong Subregion (GMS) where artemisinin-resistant *P. falciparum* (ARPf) is well established, continues to emerge [3 4 5 6 7 8 9], and has contributed to the failure of dihydroartemisinin-piperaquine (DHAPP), the first-line ACT in Cambodia [10 11 12 13].

ARPf is now at the threshold of the Indian subcontinent [14] and the great fear is that it will eventually reach Africa where it may lead to substantial reversals in malaria control achieved by the widespread use of ACTs.

The 2012 recommendation was pragmatic, taking into account the lack of detailed, carefully conducted dose-optimisation studies of PQ, the reluctance to use PQ in the GMS because of the fear of PQ-induced acute haemolytic anaemia (AHA) in G6PDd patients, the logistical impossibility of testing widely for G6PDd, and the urgency to eliminate ARPf in the GMS before it spreads to Africa. The ERG also called for more research on SLDPQ.

Single low-dose primaquine (SLDPQ) in Africa

There have been few studies of SLDPQ in Africa. Efficacy studies assessing PQ have been in G6PD normal patients and have used either DHAPP or artemether lumefantrine (AL)[15 16]. One dose ranging study of PQ in G6PD normal patients suggested that the minimum dose of PQ to produce a maximum reduction in mosquito infectivity was probably ~0.125 mg/kg [15] whereas a Chinese study suggested a PQ dose between 0.0625 and 0.125 mg/kg of PQ [1].

Studies assessing the safety of SLDPQ, have been in G6PDd and normal individuals with asymptomatic falciparum malaria; entry to such studies has required a high initial Hb concentration e.g. ≥ 11 g/dL (SAFEPRIM - NCT02174900, SAFEPRIM-II - NCT02654730). Data from the latter study were presented in 2015 but have not yet been published. They show that there were mild falls in Hb in the G6PDd individuals that was similar to G6PD normal individuals who received 0.2 or 0.4 mg/kg of PQ, but little change in the G6PDd group that received AL alone.

However, when 0.75 mg/kg of PQ was given to African children < 12 years old with one dose of sulphadoxine-pyrimethamine, those homozygous or hemizygous for G6PDd had greater falls in mean Hb than heterozygous females and those who were G6PD normal [17]. G6PDd patients with greater falls in Hb were also those who received greater mg/kg doses. Indeed, an examination of the PQ dosing regimen used shows that many children received doses in excess of 0.75 mg/kg. No PQ pharmacokinetic work (PK) was done in this study.

There is a broad consensus that dosing regimens should be simple and, in settings where dosing by weight is not possible, to have effective and tolerated drug regimens based on age. Several ACTs have both age and weight band recommendations. No age based dose regimen exists for SLDPQ but one has been proposed for Cambodia (Bob Taylor, *in press*). An age based regimen of SLDPQ for Africa would be an advance and is proposed in this study.

Pharmacology

There has been very little PQ PK work and most of this work is in adults. Modelled data from one study in healthy G6PD normal children aged 5-12 years from Papua New Guinea (PNG) found PQ PK characteristics equivalent to those in adults [18]. This is a potentially significant finding because a failure to appreciate that children < 5yrs have higher clearance rates of some antimalarial drugs compared to

adults has resulted in under dosing of sulphadoxine pyrimethamine and DHAPP [19 20]. There are no PQ PK data in young African children with *P. falciparum* malaria.

PQ has several metabolic pathways and undergoes hepatic oxidative deamination to its principal, inactive metabolite, carboxyPQ (cPQ), chiefly by monoamine oxidase (MAO) A [21 22]. Cytochrome (CYP) P450 isoenzymes 2D6, 2C19, and 3A4 are quantitatively less important but CYP 2D6 is fundamental to PQ's pharmacodynamic (PD) effects. CYP 2D6 produces oxidative metabolites (OMs) that cause acute haemolytic anaemia (AHA) [23 24 25 26 27], dose-dependent oxidation of Hb to metHb [28 29 30], and are key to PQ's antirelapse efficacy [21 31] and, probably, its gametocytocidal effect.

CYP 450 2D6 activity is under polymorphic genetic control resulting in several metabolic patterns: poor (poor efficacy, less dose-related toxicity), intermediate, extensive (normal), and ultrarapid/ultraextensive (potentially enhanced efficacy and dose-related toxicity) [32]. The genetic bases for these metabolic phenotypes are complex and their clinical predictive value unclear [33 34].

Summary

Similar to their counterparts in SE Asia, African Ministries of Health (MoH) have been reluctant to adopt SLDPQ because of PQ's toxicity, the lack of a paediatric formulation, the limited number of PQ tablet strengths and the lack of an easy to use dosing regimen [35].

This study proposal answers the WHO's call for more research on SLDPQ and will fill a number of substantial knowledge gaps in young African children, particularly as we propose using both AL and DHAPP. AL is the first line ACT in Uganda and the DRC whilst DHAPP is a favoured drug for malaria elimination because of its excellent tolerability and long half-life.

Testing DHAPP in this large trial will also allow us to compare the PK of SLDPQ in DHAPP and AL treated children. Other knowledge gaps we will fill include the: (i) tolerability of SLDPQ at the WHO recommended dose in children with high biomass falciparum infections and frequent concomitant anaemia, (ii) factors associated with the development of severe/profound anaemia, and (iii) prevalence rates of CYP 2D6 polymorphisms and their relationships to PQ PK.

2 AIMS & OBJECTIVES

The aim of this study is to provide evidence for drug policy on the safety of the WHO recommended SLDPQ regimen when used under close to routine conditions to G6PDd African children with acute uncomplicated *P. falciparum*.

2.1 Primary Objective

The primary objective will answer this research question: is a single low but gametocytocidal dose of PQ (0.25 mg/kg) safe and well tolerated when added to AL or DHAPP for treating glucose-6-phosphate dehydrogenase deficient African children with acute uncomplicated *P. falciparum* malaria?

2.2 Secondary Objectives

These are to:

- explore risk factors for developing profound or severe anaemia in this trial
- model the determinants of changes in haemoglobin concentrations over time
- characterise G6PD deficiency, other inherited blood disorders (IBDs) and the cytochrome 2D6 polymorphisms
- define the PK characteristics of PQ, cPQ, lumefantrine and piperaquine
- determine changes in gametocyte carriage over time
- measure the parasite clearance over time and the D42 cure rate

- assess the test characteristics of a G6PD Biosensor

3 STUDY METHODS

3.1 Study sites

The study will take place in Kinshasa in the Democratic Republic of Congo and in Uganda.

In Kinshasa, the Kinshasa Mahidol Oxford Tropical Medicine Research unit (KIMORU) has a clinical laboratory, a 15 bedded ward, and a full-time, GCP trained, research team of doctors, nurses and laboratory technicians. Malaria transmission is intense and KIMORU sees ~10,000 outpatient malaria cases/year from the Kinshasa conurbation, mostly children under five years of age.

The Mbale Regional Referral Hospital is situated in eastern Uganda in an area of perennial hyperendemic malaria. It serves a wide, predominantly rural, catchment area. Annual admissions to the paediatric ward are ~20,000 and approximately 140 patients per day are seen in outpatients of whom some 110 have malaria (Olupot-Olupot, unpublished)

3.2 Study design

This is a randomised, multicentre, parallel-group safety trial of SLDPQ when combined with the first-line antimalarial therapy, AL, or DHAPP for treating acute uncomplicated *P. falciparum* malaria in G6PDd and G6PD normal African children. The trial is double blind with respect to SLDPQ but AL and DHAPP are open.

The trial will be stratified by G6PD status based on the results of a G6PD rapid diagnostic test (RDT)*:

- a G6PDd group that will be randomised to AL or DHAPP plus SLDPQ or placebo, and
- a G6PD normal group that will be randomised to AL or DHAPP plus SLDPQ or placebo

* we will use the AccessBio G6PD RDT. If a site also wishes, the fluorescent spot test (FST) can be used. Where there are discrepant FST RDT results, preference will be given to the FST.

3.3 Primary Endpoint

The primary outcome is the proportion of patients who, within the first 21 days, develop either:

- profound anaemia [Hb concentration < 4g/dL] or
- severe anaemia (Hb <5g/dL) with severity features e.g. impaired consciousness or deep breathing.

3.4 Secondary endpoints

The secondary endpoints are to determine/measure/assess:

- Haemoglobin concentrations over time:
    - Fractional change in haemoglobin on Day 7 *vs.* baseline
    - Proportion of patients with a fractional change ≥ 25%
    - Time to nadir Hb concentration
- Determinants of changes in Hb concentration over time
- G6PD genotype (hemizygous male, homo-, heterozygous female, normal and selected G6PD variants e.g. G6PD A- 202 mutation
- G6PD enzyme activity

- Genotypes of other inherited blood disorders
- Incidence of adverse events
- Pharmacokinetic characteristics of PQ, carboxyPQ, lumefantrine and piperaquine
- CYP 2D6 genotypes
- Asexual parasitaemia clearance time and half life
- Therapeutic efficacy of AL and DHAPP using the WHO criteria (Appendix 3)
- Gametocytaemia over time
- Proportion of patients with gametocytes over time

3.5 Inclusion criteria

- Age ≥ 6 months to <12 years
- Presentation with:
  - clinically uncomplicated disease
  - fever (≥ 37.5°C aural) or history of fever within the previous 72 hours, and
  - a positive malaria slide for *P. falciparum* (mono or mixed infection) of any parasitaemia (Kinshasa only)
- Informed consent provided by patient or relative/legal guardian

3.6 Exclusion Criteria

The patient/subject will not enter the study if ANY of the following apply:

- Malaria danger signs, sign(s) of severe malaria, or decompensated anaemia, including:
  - An inability to take or retain fluids or oral medications
  - Confusion
  - Prostration
  - Convulsions
  - Respiratory distress
  - Passing of red or cola-coloured urine (putative “blackwater fever”)
- Hb <6 g/dL
- A comorbid illness that requires treatment in hospital (physician’s judgement)
- Patients on drugs known to cause haemolysis in G6PDd e.g. dapsone, nalidixic acid
- Known to be allergic to PQ, AL, or DHAPP
- Previous enrolment in the current trial or current enrolment in another trial

3.7 Initial assessment at outpatients or clinic

Potentially eligible children (i.e. children with a febrile illness) will be identified by the nurse or clinician on duty in outpatients in Uganda and at the KIMORU centre in Kinshasa and will be assessed for suitability for the study.

3.7.1 Uganda

A member of the trial team will then perform a rapid structured assessment to initially exclude any child with clinical symptoms or signs indicating the need for hospitalisation (an exclusion criterion). Children with uncomplicated febrile illness will be registered in the eligibility screening log.

In order to use resources efficiently, reduce wasted time for the caregivers and children, and limit the number of finger prick blood tests on children, a two stage process is proposed for the screening and enrolment of our study subjects.

First, caregivers or guardians of children who are potentially eligible will be approached by a member of the trial team to invite them to join a clinical trial and explain very briefly this may involve hospital admission for 1-4 days and extra blood tests. Caregivers could at this stage could ‘opt out’ or ask for more information.

If they agree or want more details then the member of the trial team would explain that the study would screen children for malaria using malaria RDT and a haemoglobin level (both are routine tests) and a RDT for G6PD deficiency (not a routine test).

All these would require a single ‘finger prick’ specimen. This would establish if they have malaria and a relevant inclusion criterion (Hb ≥ 6 g/dL) and their G6PD status. It is anticipated that this process will take approximately 5 minutes of explanation and up to approximately 10 minutes for the screening blood tests.

Once the caregiver/legal guardian agrees to **consider** entry into the study, we will conduct finger prick sample for the screening blood tests:

- Malaria RDT
- Hb concentration (HemoCue)
- G6PD status using the AccessBio RDT (CareStart®)

Following the results of these tests, we will know if a child is suitable to join the study. If so and the caregivers / guardians still wish to proceed, a patient information sheet and consent form will be given to the caregivers/guardians to read and ask questions.

A research team clinician will oversee the consent process and answer questions. The caregivers/guardians can either decline to join the study or sign the consent form to proceed to the more detailed prestudy enrolment assessment.

3.7.2 Kinshasa

In Kinshasa, out-patients with fever present directly to the KIMORU unit where they are tested routinely for malaria by RDT, and have a Hb concentration measured. If they are RDT positive, a blood film is done and if positive, they receive standard antimalarial treatment.

When a study takes places at KIMORU, a study nurse talks to the waiting caregivers of the potential patients to inform them that a study is taking place. If a caregiver/legal guardian expresses an interest to join the study, then verbal consent will be obtained to perform:

- malaria RDT
- a malaria film if the RDT is positive
- Hb concentration (HemoCue)
- G6PD status using the AccessBio RDT and/or FST

After children are seen by the study doctor, and the inclusion and exclusion criteria confirmed, a study nurse will proceed with the informed consent process with the caregivers/legal guardian.

3.7.3 Screening failure / caregiver decline

If the screening process finds that the patient is not suitable to join the study or the caregivers or guardians decline to join the study, the child will be treated for malaria according to local guidelines.

3.8 Enrolment into the study

Once caregivers have signed the consent form to join the study, a study (randomisation) number will be given and the following study assessments will be done:

- History of current illness, past medical, family and drug histories
- Symptoms checklist
- Detailed physical examination
- Baseline methaemoglobinaemia (by oximetry)
- Finger prick sample for:
  - Hb and haematocrit (HCT) concentration
  - Malaria blood film (H0)
  - Blood film for reticulocyte count
  - Blood spots for
  - parasite genotype, molecular markers of resistance
  - gametocyte qPCR
- Venous blood for:
  - Full blood count & reticulocyte count (if a suitable machine is available)
  - G6PD enzyme activity by gold standard spectrophotometric quantitative assay
  - G6PD & CYP 2D6 genotyping
  - Routine biochemistry, including total & unconjugated bilirubin, LDH, haptoglobin and creatinine
  - PQ, lumefantrine and piperaquine concentrations (on a subset)

3.9 G6PD status

We will assess G6PD status using the AccessBio CareStart™ RDT at the time of screening. This RDT has high sensitivity (>95%) for detecting enzyme activities ≤ 3.6 U/g Hb which represent ~30% of normal activity in different settings [36] [37] [38]

As mentioned above, sites have the option to use the FST and if the RDT FST results are discrepant, preference will be given to the FST.

Therefore, the G6PDd group will consist of mostly hemizygous males, some homozygous females and a few heterozygous females with low G6PD enzyme activities. The RDT diagnosed “normal” group, will comprise mostly G6PD normal males and females and a G6PD heterozygous females with intermediate enzymatic activity [39] [40].

The G6PD results will be used to stratify the randomisation and keep a tally of recruitment numbers by G6PD status.

3.10 Drug allocation and randomisation

The study has four dosing groups for the G6PDds and four dosing groups for the G6PD normals:

- AL + SLDPQ

- AL + SLDPQ placebo
- DHAPP + SLDPQ, and
- DHAPP + SLDPQ placebo.

A computer generated randomisation list will be generated for each site:

- one list for G6PDd patients and
- one list for G6PD normal patients.

The treatment allocation will be placed in a sealed opaque envelope which will be opened after a study (randomisation) number is given. The treatment allocation will describe the ACT to be given and the number of the PQ/placebo pack.

3.10.1 Primaquine/placebo

Owing to the low prevalence of G6PDd (~12%), the recruitment rates for the G6PDd arms will be much slower than those of the G6PD normal arms; therefore, the G6PD normals will reach their target sample size much earlier.

This will be adjusted for because there will be separate G6PDd and G6PD normal randomisation lists; this is called stratified block randomisation and is shown in Figure 1.

Figure 1. The stratified block randomisation scheme showing the final numbers to be allocated to each treatment.

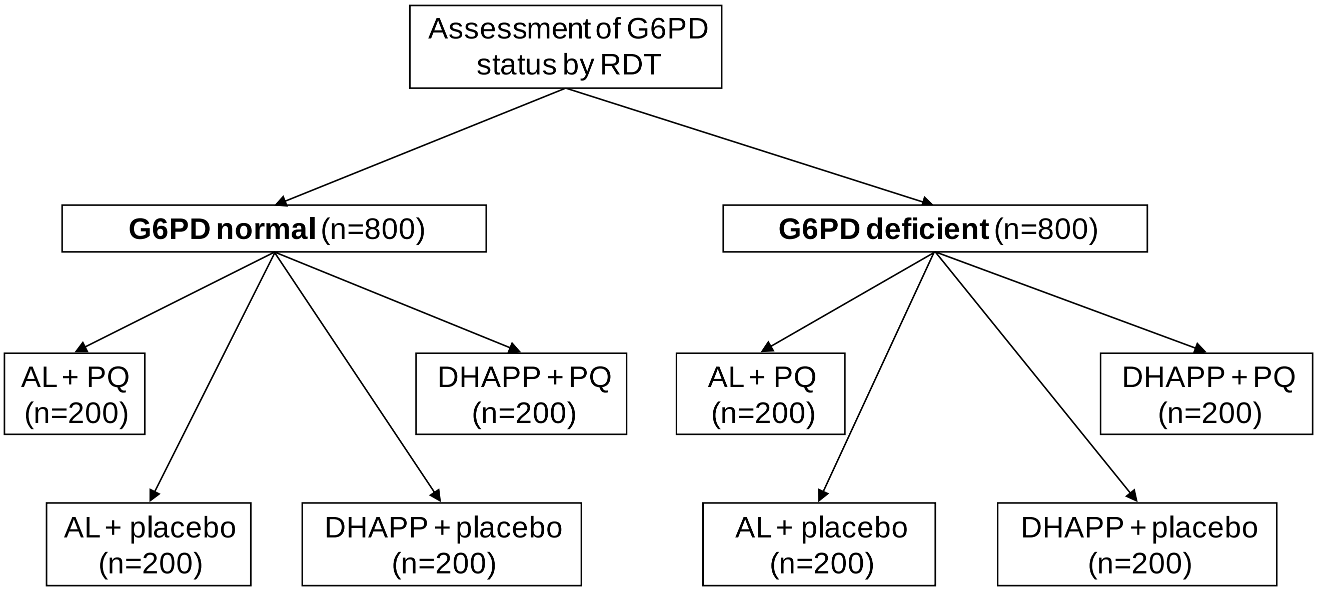

3.10.2 Primaquine/placebo administration

PQ/placebo dosing will be based on age. There will be four blister packs labelled A, B, C and D for the four age groups (see Section 5.3). Blisters A and B will contain 2.5 mg of PQ/placebo and C and D will contain 5 and 7.5 mg of PQ/placebo, respectively.

The PQ/placebo blister packs will be labelled sequentially to match the randomisation sequence for that age dosing band and will be allocated to patients in numerical order. Therefore, each patient will have a unique drug number as well as a unique subject number.

An example of the numbering is shown below for age band A, G6PDd: A-D-001

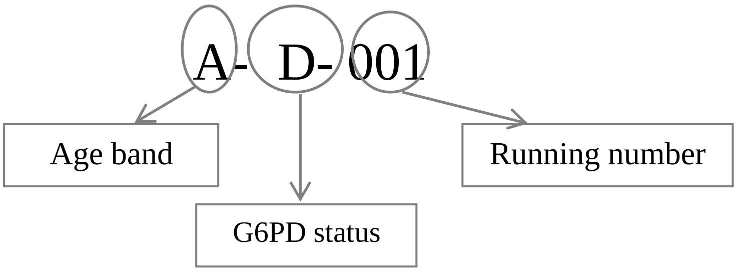

The running number is 001 for the first patient, 002 for the second patient etc. We will discuss the drug packaging and randomisation system with Bilcare (the packaging company) and then decide on/fine tune the final labelling and randomisation scheme.

The allocation of AL or DHAPP and PQ/placebo will be organised at each site in order to minimise the chances of an error in drug allocation. Accordingly, a detailed Randomisation / Drug management guideline will be written.

3.10.3 Balancing recruitment numbers

Data from Kinshasa and Uganda show that children aged 6-11 months and those aged 5-11 years are relatively under represented. We will, therefore, review periodically the distribution of ages of our recruited patients from both sites combined and will aim to recruit a total of at least:

- 80 children aged 6-11m and
- 120 children aged 5-11 years.

This may require a temporary suspension of the recruitment of some age group as the trial proceeds.

3.10.4 Option to dose primaquine/placebo on Day 1

Data show that early vomiting within one hour can affect a significant minority of patients when treated for falciparum malaria. Children under five are most affected and rates within the under 5s are inversely related to age [41].

Pooled data from the World Wide Antimalarial Resistance Network show rates of early vomiting with DHAPP ranging from 3-18% on Day 0 compared to 1-9% on Day 1. Corresponding rates in AL treated children aged < 5 are from 2.3-7.3% and 0.5-6%. These data are consistent with the experience from KIMORU [42].

Initially, we will dose all children on Day 0 and record rates of early vomiting (indeed this is an adverse event of interest – see Section 8).

When approximately half of the patients have been recruited, we will analyse the data and consider whether we wish to dose PQ/placebo on Day 1.

3.10.5 Patients not completing treatment

Patients may not complete their treatment for the following reasons:

- persistent vomiting of study drugs. A patient who vomits his/her AL or DHAPP +/- PQ/placebo within one hour and also the repeat dose/s will be given rescue treatment and when better will continue follow up to D42 (see Section 6).
- the development of an adverse / serious adverse event necessitating the early stopping of study drugs; these patients will remain in the study to D42
- the PI withdraws the patient from the study before treatment is completed (see Section 4.1).

3.10.6 Admission to hospital

At the KIMORU research centre in Kinshasa, study patients are routinely admitted for at least three days or longer according to their clinical course.

In Uganda, all caretakers/guardians will be invited to allow their child to be admitted to hospital for at least 48 hours for close monitoring and to receive directly observed therapy for all doses of their treatment. Children of caretakers/guardians who decline this invitation will be followed up as outpatients; this will include daily visits to the hospital so as to record clinical observations and monitor their treatment.

This decision has been taken mostly because caretakers/guardians prefer their children to be admitted.

Hb data will be continue to be sent weekly to the Data Monitoring and Ethics Committee (DMEC) by both sites.

3.10.7 Follow up

After discharge from hospital, children will be reviewed as outpatients up to D42 (Appendix 1).

At each visit, the following will be done:

- clinical symptom checklist by the doctor/nurse, including vital signs
- Giemsa stained malaria blood films
- Hb & HCT or full blood count

At certain visits only, the following will be done:

- blood films for reticulocyte count
- filter paper blots for parasite genotype and qPCR for gametocytes
- venous blood samples routine biochemistry and lumefantrine blood concentration
- patients will be asked to return to the clinic at any other time if they feel unwell or develop a fever.

3.10.8 Blood volume

The blood volumes per test are detailed in the schedule. The total volume of blood, including the samples for PK, is approximately 34.5 mL but allowing for spillage and/or repeat samples we seek permission for a total blood volume of 38 mL.

In patients not having intense PK sampling for primaquine, the total blood volume will be 6 mL less i.e. 29 mL but permission will be sought for 30 mL in Uganda and Kinshasa.

3.10.9 Duration of Study

We expect the study to start within the first three months of 2017 and to complete recruitment and follow up within 24 months. However, we seek permission for 30 months of field time.

4 WITHDRAWAL & LOST TO FOLLOW UP

4.1 PI withdrawal

The PI may withdraw the patient e.g. if the study subject is poorly compliant with the study schedule or the research physicians think it is unsuitable or unsafe for the patient to continue in the study. In this scenario, follow up will cease but permission to use patient data up to the point of withdrawal will be sought.

4.2 Patient withdrawal

Study patients are considered withdrawn from the study if:

- Their caregivers/legal guardian withdraw consent for study continuation

This means no more study investigations will be done after that point.

The consent form has an option regarding the use of the child's data if he or she is withdrawn from the study by the care giver/legal guardian.

Data will not be used in the analysis unless their caregiver/legal guardian has given written consent that data can be used.

4.3 Loss to follow-up

Every effort will be made to trace patients not returning to the centre for follow-up visits. Home visits will be carried out by a team member for cases who do not attend for follow-up.

A patient will be considered lost to follow up if she/he does not come for follow up and cannot be found by D42 of their schedule. Missing some follow up visits but reappearing before or on study D42 is not considered lost to follow up.

The time-window allowed for routine follow up visits:

- study day7 is +1 day
- study days 14 – 42 is -1, +2 days

4.4 Unscheduled visits

Patients presenting to the clinic with a fever or other symptoms on unscheduled days will be assessed by the study physician. Their temperature will be recorded, a blood smear will be made and the Hb measured by HemoCue. Patients will be treated as clinically indicated and will continue study procedures. Data from unscheduled visits will be recorded in the CRF and will be analysed.

4.5 Protocol deviations & violations

A protocol deviation is a departure from the approved protocol that does ***not*** result in harm to the trial patients whereas a violation is potentially harmful.

Protocol deviations include:

- the patient took an antimalarial drug not prescribed by the research team
- giving PQ at the wrong time
- Hb not being measured as per schedule
- delay in reporting SAE
- consent form not properly signed

Protocol violations include:

- enrolment violation – a patient who was wrongly included in the study
- patient was enrolled without signing the consent form
- the wrong dose of study drug/s was/were given

- misreading a negative malaria as positive resulting in patients receiving rescue treatment
- the patient was given (either by the research team or from another source) a drug (other than PQ/placebo) known to cause haemolysis in G6PDd

Protocol deviations and violations will be recorded on the protocol deviation/violation form and reported to the DMEC.

5 STUDY DRUGS

5.1 Artemether lumefantrine

AL is the recommended first line drug for falciparum malaria in DRC and Uganda.

GMP manufactured AL will be obtained from a reliable manufacturer or official supplier either locally or externally and imported. If imported all necessary documentation will be obtained (e.g. certificate of analysis, etc). AL will be dosed by weight, in accordance with the manufacturer's recommendation. One tablet of dispersible and non-dispersible AL contains 20 mg of artemether and 120 mg of lumefantrine.

AL is dose twice daily. On the first day of treatment the doses are separated by 8 hours but by 12h on the second and third days of treatment. The total course is 6 doses.

Weight in kg	Number of tablets / dose	Total number of tablets / day	Total number of tablets / course
5 - <15	1	2	6
15 - < 25	2	4	12
25 - < 35	3	6	18
≥ 35	4	8	24

5.2 Dihydroartemisinin piperaquine

DHAPP has been extensively used in Africa [43] [44] and Asia [45] and the Sigma Tau formulation is registered in several African countries and was registered in the European Union in 2011.

GMP produced DHAPP will be obtained from a reliable source and, if imported, all necessary documentation will be obtained. DHAPP will be dosed by weight. The 2015 WHO guidelines now recommend a dose of ≥ 2.5 mg/kg/day of DHA in children who weigh < 25 kg (http://www.who.int/malaria/publications/atoz/9789241549127/en/).

There are three DHAPP tablet strengths, all in mgs:

- DHA 20 PP 160
- DHA 40 PP 320
- DHA 80 PP 640

The teams will modify the WHO dosing chart according to the tablet strengths they are able to obtain in Kinshasa and Uganda.

Table 1. DHAPP dosing table from the WHO.

Body weight (kg)	Dihydroartemisinin + piperaquine dose (mg) given daily for 3 days
5 to < 8	20 + 160
8 to < 11	30 + 240
11 to < 17	40 + 320
17 to < 25	60 + 480
25 to < 36	80 + 640
36 to < 60	120 + 960
60 < 80	160 + 1280
>80	200 + 1600

5.3 Primaquine/placebo

PQ/placebo (2.5, 5, 7.5 mg PQ base) will be manufactured for this trial by Centurion Remedies, PVT Ltd, Gorwa, India. This is a WHO certified manufacturing facility. Bilcare Ltd, Pune, India, will blister pack the PQ/placebo and will manage their export from India to the DRC and Uganda, in accordance with all applicable drug regulations.

Bilcare Ltd has already supplied quality, GMP manufactured PQ/placebo for MORU for another large trial (NCT01814683). The batch of PQ/placebo used for this trial will be quality controlled and a certificate of analysis will be supplied by Bilcare Ltd.

Dosing will be by age. The target dose of PQ is 0.25 mg/kg with a therapeutic dosing range of 0.15 - 0.4 mg/kg given once with the first dose of AL or DHAPP [1]. Children will receive doses ranging from 0.1 - 0.46 mg/kg. A detailed breakdown of the mg/kg dose that a child will receive is shown in Table 2.

For young children who cannot swallow, the PQ/placebo tablet will be dissolved in 5 mL of sugar water (or similar solution depending on local practice) and the dose administered. The 2.5 mg PQ/placebo tablet will be used for children aged 6-11m but they will be given 2.5 mL of suspension and so will receive 1.25 mg of PQ/placebo. Guidelines on the practicalities of drug administration will be written.

Table 2. Dosing table by age of PQ showing the mg/kg dose received.

Age	Primaquine dose in mg base	minimum	1st quartile	median	3rd quartile	Maximum
6-11m	1.25	0.1	0.15	0.16	0.18	0.3
1y	2.5	0.16	0.25	0.27	0.3	0.46
2y	2.5	0.14	0.2	0.22	0.25	0.37
3y	2.5	0.12	0.17	0.19	0.21	0.32
4y	2.5	0.11	0.16	0.17	0.19	0.29
5y	2.5	0.1	0.15	0.16	0.18	0.25
6y	5	0.17	0.26	0.29	0.32	0.44
7y	5	0.16	0.24	0.26	0.29	0.39
8y	5	0.14	0.21	0.24	0.26	0.34

9y	5	0.12	0.2	0.21	0.24	0.3
10y	7.5	0.17	0.26	0.29	0.32	0.4
11y	7.5	0.16	0.24	0.27	0.29	0.36

5.4 Drug supervision

All doses of study drugs will be administered by research nurses. A small snack or milk will be given with every dose to reduce the risk of PQ induced abdominal pain and to increase the absorption of lumefantrine and piperaquine.

The study patients will be observed for 60 min. Any patient who vomits during this observation period will be retreated with the same dose of AL, DHAPP and PQ/placebo (and observed for an additional 30 min).

If the vomiting occurs between 31 and 60 minutes, patients will receive an additional:

- half dose of AL or DHAPP
- half dose of PQ/placebo

If the patient vomits again, oral treatment will be stopped and parenteral rescue therapy will be given (See Section 6).

5.5 Concomitant drugs that must not be prescribed

Patients and guardians will be asked not to visit local pharmacies, doctors and drug vendors to buy drugs or herbal medicines, in case they take drugs that can cause AHA in G6PDd.

Below is a list of drugs that may cause AHA in G6PDd and are contraindicated in this study:

Potentially haemolytic drugs are listed below.

- Dapsone
- Sulphonamides
- Methylene blue
- Nalidixic acid
- Nitrofurantoin
- Niridazole
- Aspirin
- Traditional medicines

The following drugs may have a possible risk of haemolysis:

- Ciprofloxacin, norfloxacin
- Chloroquine

The following drugs are considered to be of doubtful risk of haemolysis:

- Chloramphenicol
- Quinine

5.6 Concomitant drugs that are allowed

Febrile patients can be treated with paracetamol (acetaminophen). During follow-up, infections other than malaria may require the administration of medicines with antimalarial activity. This is allowed.

All drugs that are taken by patients will be noted on the Concomitant drug form and any details of adverse events (AEs) noted on the AE CRF.

5.7 Concomitant drugs that may interfere with primaquine

The following drugs may increase the exposure of PQ, piperaquine and lumefantrine metabolism by inhibiting the cytochrome (CYP) 450 and should not be prescribed by the research team unless clinically essential:

- Ketoconazole, itraconazole
- Cimetidine
- Grapefruit juice
- Erythromycin
- Ritonavir

The following drugs may decrease the exposure of PQ, piperaquine and lumefantrine metabolism by inducing CYP 450. They should not be prescribed by the research team unless clinically essential:

- Barbiturates
- Carbamazepine
- Phenytoin
- Rifampicin
- Macrolide antibiotics
- Glucocorticoids

The following drug classes are metabolised by CYP 2D6 and may compete with PQ[46]. They should not be prescribed by the research team unless clinically essential:

- tricyclic antidepressants e.g. amitriptyline
- major tranquilisers: haloperidol
- atypical antipsychotics: risperidone
- selective serotonin reuptake inhibitors: paroxetine
- beta blockers e.g. carvedilol, metoprolol
- opiates: codeine, tramadol
- antimalarials: pyronaridine

The following drugs are CYP2D6 inhibitors and may reduce the concentrations of PQ's active oxidative metabolites. They should not be prescribed by the research team unless clinically essential:

- amiodarone
- cimetidine
- diphenhydramine, terbinafine
- fluoxetine, paroxetine

- quinidine
- ritonavir

6 RESCUE TREATMENT

6.1 Parenteral rescue treatment

Parenteral rescue treatment will consist of intravenous or intramuscular artesunate and will be given for the following clinical indications:

- Persistent vomiting of study drugs
- The development of severe malaria/danger signs at any time during the study.

Parenteral artesunate is the preferred rescue treatment, followed by artemether. Quinine is the third choice.

6.2 Oral rescue treatment

Patients will be treated with oral amodiaquine-artesunate or a different ACT according to National Guidelines if they:

- fail to either clear their *P. falciparum* parasites within the first 7 days or
- develop asymptomatic or symptomatic recurrent malaria during the follow-up

All patients who require rescue treatment will be followed-up until study Day 42.

7 ASSESSMENTS

These are outlined in the schedule (Appendix 1). Further details are given below.

7.1 Clinical evaluations

7.1.1 History, symptoms & examination

These will follow a standard format and data will be entered onto the case record form (CRF). The symptom checklist will be readministered on Days 3, 7, 14 etc to D42. Vital signs (pulse, blood pressure, respiratory rate and temperature) will be recorded 12 hourly in hospital and done at each outpatient visit. The aural or axillary temperature will be taken.

Body weight will be recorded at screening to the nearest kilogram using a suitable weighing scale. The scales will be properly calibrated.

7.1.2 Reading malaria blood films

Blood films will be prepared at baseline, 6 and 12 hours and then every 12 hours until 2 consecutive negative blood films are observed (inpatients only).

Asexual and sexual parasites will be identified and counted by microscopy (magnification of 1000×) on Giemsa-stained thin and thick films.

Parasitaemia will be measured on a thin blood film if more than 200 parasites per 10 fields on a thick film are seen: 2000 RBCs will be read and the number of parasitised infected RBCs noted.

Below this threshold, the parasitaemia will be counted per 500 WBC on the thick film.

Slides are considered negative after examination of at least 200 high-power microscopy fields.

The final parasitaemia is calculated as follows:

- <u>Thin film: N parasites/2000 red blood cell on the thin smear x Hct x 125.6</u>

2

- Thick film: N parasites/500 white blood cells on the thick smear x 16

Slides will be cross checked blinded by the research team, as follows:

- 10% of all D0 slides
- all positive slides on D3 and D7
- 10% of negative slides on D3 to D28.

Two qualified microscopists will read all the slides independently. Blood smears with discordant results:

- different parasite species
- a difference in parasite density of > 50%
- positive/negative slide

will be re-examined by a third, independent microscopist, and parasite density will be calculated by averaging the two closest counts.

7.1.3 Gametocyte quantification by qPCR

These will be measured on Days 0, 3, 7, 14, 21, 28, 35 and 42. A small volume of whole blood (0.1 mL is preferred) from the finger stick will be placed in a cryovial and a suitable preservative will be added such as RNA protect or trizole. Gametocytaemia will be measured using a quantitative nucleic acid sequence based assay to detect male (Pfs230p) and female (Pfs25) gametocytes on a subset of patients [47]. Samples will be analysed centrally at the KEMRI unit in Kilifi in Kenya and, possibly also, at MORU in Thailand.

7.1.4 Blood film for reticulocyte count

A thin blood film will be stained with cresyl blue and read by an experienced microscopist. A SOP will be written for the preparations of these examinations in the field.

7.1.5 Full blood count, HemoCue & HCT measurements

A full blood count will be measured on D0, 3, 7 and 28. Thereafter, Hb concentrations will be measured daily for inpatients and at every outpatient visit using the HemoCue system.

We will assess the accuracy of a simple to use, field adapted method for estimating the Hb concentration – the WHO (Copack) Haemoglobin Colour card [48]. Blood will be taken at baseline, each follow up visit and when patients have recurrent parasitaemia. This will be done in Kinshasa only.

The HCT will be measured using a microcentrifuge.

7.1.6 Standard spectrophotometric quantification of G6PD enzyme activity

This test requires a spectrophotometer and will be done using a suitable kit e.g. the G6PD enzyme assay from Trinity Biotech, USA or R & D diagnostics, Greece. This method reports the G6PD enzyme results as a change in absorbance over time. This value is normalised using the Hb concentration to give an enzymatic activity in U/g Hb. It will be done on D0. This method is considered the gold standard and will be used to assess the performance of the G6PD RDT and the G6PD Biosensor.

7.1.7 G6PD Biosensor

We will assess the test characteristics of a G6PD quantitative biosensor. This point of care test can measure the G6PD activity using a drop of blood in few minutes. We will compare it to the standard spectrophotometric quantitative G6PD test on D0.

7.1.8 The G6PD rapid diagnostic test

As described in Section 3.9, we plan to use the AccessBio G6PD RDT and assess its characteristics compared to the gold standard spectrophotometric method.

7.1.9 G6PD genotype

The A^- Glucose-6-Phosphate Dehydrogenase deficiency defined by G6PD 376 (rs) and G6PD 202 (rs) will be characterised from genomic DNA using the Agena MassArray IPLEX platform as previously described [49].

7.1.10 Characterising sickle cell disease and alpha thalassaemia

Genotyping for sickle cell disease and the common African form of alpha (α) thalassemia, caused by a 3.7-kilobase pair deletion in the α-globin gene, will be conducted by PCR, consistent with published methods [50 51].

Children with one $beta^S$ (β^S) mutation of the Hb gene will be classed as sickle cell trait (HbS) whereas homozygotes will be classed as sickle cell disease (HbSS). For α-thalassemia, children with a single α-(−α / αα) or two α-globin deletions (−α / −α) will be classed as homozygote and heterozygote for α-thalassemia, respectively.

7.1.11 CYP 2D6 genotype

Several commercially available methods are available that use PCR platforms. We will select the specific platform at study end e.g. the Luminex system (Luminex® Corporation, USA). Allelic variation categories assayed by this kit include DNA sequences encoding normal metabolic function (*1, *2, and *35), reduced metabolic function (*9, *10, *17, *29, and *41), and non-functional (*3, *4, *5 –for whole-gene deletion, *6, *7, *8, *11, and *15), including DUP for duplication. The data will be analysed automatically using the xTAG Data Analysis Software ™ to record the CYP2D6 allelic variant. A decision on which PCR system to use will be made nearer the time of analysis.

7.1.12 Percutaneous measurement of methaemoglobin

PQ causes a dose dependent rise in blood methaemoglobin (metHb). Daily PQ doses of 22.5-30 mg may produce metHb values of up to 18% which are well tolerated in healthy people [52 53]. MetHb concentrations fall to normal concentrations within 7 to 14 days.

In Cambodia, 0.75 mg/kg of PQ base resulted in a modest increase of metHb – the maximum rise was 4.9% [54]. The upper limit of normal is between 2-3%. Therefore, we do not expect that PQ in our study to result in clinically significant metHbaemia.

A haemoximeter (e.g. Masimo Rad57 upgraded) will be used to measure the methaemoglobin content.

The following is a guide to the symptoms associated with methaemoglobinaemia.

- Up to 2-3% Normal
- Less than 10% metHb - No symptoms
- 10-20% metHb - Skin discoloration only (most notably on mucus membranes)
- 20-30% metHb - Anxiety, headache, dyspnoea on exertion
- 30-50% metHb - Fatigue, confusion, dizziness, tachypnoea, palpitations
- 50-70% metHb - Coma, seizures, arrhythmias, acidosis
- Greater than 70% metHb - Death

7.1.13 Specimen labelling

Specimens and specimen tubes will be labelled as follows.

A label will be stuck onto the tube or specimen and the research team member will write:

- subject initials
- subject ID (study number)
- date and time of sample collection

- study day
- sample type e.g. plasma, serum etc.

For samples that will be sent out of the site, a bar code system will be used. One barcode label will be stuck to the sample tube / specimen and another bar code label to the sample log.

7.2 Primaquine, lumefantrine and piperaquine concentrations

There are no PQ/cPQ PK data in children <5 years so data from this trial will be crucial to determine whether the age based regimen in this study is suitable for children in this age group and allow us to compare PQ and cPQ exposures as a function of age and the ACT given.

The pharmacokinetics of PQ and cPQ will be characterised by non-compartmental analysis from blood samples at fixed time points. PK sampling will be done in Kinshasa and Uganda. The number of patients who will be sampled at each site will be 300:

- AL treated children aged < 5y (n=100)
- AL treated children aged 5-11 years (n=50)
- DHAPP treated children aged < 5y (n=100)
- DHAPP treated children aged 5-11 years (n=50)

Therefore, a total of 600 patients will be sampled. Approximately half will have received PQ and the other half placebo. Thus, the total number of patients with PQ PK data will be 300. We will keep a tally of patient numbers and once the required number of patients has been sampled we will stop the PK. Given the relative lack of children aged 5-11 years, we may not reach the sample size for this group.

For those children who received placebo, we will analyse the lumefantrine and piperaquine concentrations in the first 24 hours. We will insert an intravenous catheter at enrolment in children that will remain in place for 24 hours and draw up to a maximum of 8 samples per child (less if demanded by the local ethics committees) at fixed time points. Given the uncertainty of the outcome of the ethical review, we will select eight time points from this schedule: 0, 1, 1.5, 2, 3, 4, 6, 8, 10, 12, 24h.

Participation in this PQ PK sampling regimen is optional in Uganda but is considered part of the study in Kinshasa.

A venous blood sample for lumefantrine and piperaquine concentrations will be taken on D0, D3, D7, and D28

The sample size for lumefantrine is 200 – ~100 who will receive PQ and ~100 who will receive placebo. The sample size for piperaquine is also 200 with a 50-50 PQ-placebo split.

Comparing the D7 concentrations of both drugs between the PQ and placebo arms will determine whether PQ has affected their dispositions. Both lumefantrine and piperaquine D7 concentrations are valuable predictors of treatment failure in *P. falciparum* [55 56].

All PK samples will be analysed at the MORU PK laboratory in Thailand using MCMS / MS.

7.3 Sample Shipment

Any samples that cannot be analysed locally will be shipped to suitable laboratories for analysis in Thailand (e.g. MORU PK laboratory), Kenya (KEMRI-WT laboratories in Kilifi), the USA (PATH, Seattle), and the United Kingdom (e.g. Sanger Institute). All relevant shipping and local and international regulatory regulations will be followed.

8 SAFETY MONITORING & ADVERSE EVENTS

Safety reporting will be done in accordance with MRC GCP guidelines. During the study, we will collect and record on the CRF symptoms, physical signs and laboratory findings. Data collection starts at study enrolment and finishes after the Day 42 follow up.

8.1 Adverse events & severity

An adverse event (AE) is any undesirable event (symptom, sign, laboratory result) that occurs to a study participant during the course of the study; that is, from the time of study drug administration until study end. An AE may or may not be related to the study drugs, a concomitant drug or a study procedure.

An AE can be absent at baseline and newly develops or was present at baseline and worsens.

We will use the NIH Division of Microbiology and Infectious Diseases (DMID) toxicity table to grade adverse events from 1 to 4.

8.2 Relatedness of Adverse Event to study drugs

Any AE that occurs will be assessed by the team physician to determine the relationship between the AE and the study drug/s. because two drugs are being used in this study, the AE may be related to one or both drugs.

This drug-AE relationship will be graded as follows:

- Unrelated: clearly not related to the study agent
- Unlikely related: doubtfully related to the study agent
- Possibly related: may be related to the study agent
- Probably related: likely related to the study agent
- Definitely related: clearly related to the study agent

8.3 Adverse Event Recording

In this study, all adverse events of all severities will be recorded.

At each visit, study participants/their caregiver/legal guardian will be asked the open question “How is your child today?” Any reported symptoms will be recorded in the medical records.

On certain study days (see study schedule in Appendix 1), a symptom questionnaire will also be administered, entered into the database and analysed. If the research team physician thinks these symptoms require further evaluation, this will be done.

The investigator is responsible for the detection and documentation of events meeting the criteria and definition of an adverse event (AE) or serious adverse event (SAE).

All SAEs and AEs will be promptly documented from the moment of inclusion in the study to discontinuation of the patient from study participation. Any events occurring between screening and randomization will be considered as baseline, preexisting conditions.

All adverse events must be recorded in the AE/SAE CRF. To avoid colloquial expressions, the adverse event should be reported in standard medical terminology. Whenever possible, the adverse event should be evaluated and reported as a diagnosis rather than as individual signs or symptoms. If a definitive diagnosis is not possible, the individual symptoms and signs should be recorded. Whenever possible, the aetiology of the abnormal findings will be documented on the CRF. Any additional relevant laboratory results obtained by the Investigator during the course of this study will be recorded on the CRF.

If the event meets the criteria for "serious", the SAE must be reported to the PAC safety team within 24 hours from the time that the event was identified. If further data is required, additional documentation can be submitted. All SAEs must be followed until resolution, or until the SAE is deemed permanent or leads to death.

8.4 Patient Management of Adverse Events

All AEs will be treated as clinically indicated and concomitant treatment recorded on the Concomitant Medication CRF. If necessary, participants will be referred for specialist care.

The research team will treat and pay for minor AEs or any AE that requires hospital management that is clearly related to the study drug/s e.g. haemolysis requiring a blood transfusion.

All patients will be followed up until the AE resolved or stabilised even if this occurs past study D42.

This includes patients who withdraw themselves or have been withdrawn from the study by the PI.

8.5 Serious Adverse Events

In this study, an AE is a serious AE if it results in any of the following outcomes:

- Death
- Life-threatening event – this means that the patient was at immediate risk of death at the time of the event and required immediate medical intervention. It does not refer to an event which hypothetically might have caused death if it were more severe.
- Prolongation of existing hospitalisation or re-hospitalisation once discharged.
- Persistent or significant disability/incapacity
- An important medical event that may not be immediately life-threatening or result in death or hospitalization but may jeopardize the patient or may require intervention to prevent one of the other outcomes listed in the definition above.

All SAEs will be recorded on the SAE CRF.

8.6 Expedited Reporting of Serious Adverse Events

Expedited reporting means the rapid reporting of SAEs.

All SAEs, irrespective of their relationship to the study drugs, will be reported within 24 hours to:

- the study PI, Pr. NPJ Day: nickd@tropmedres.ac
- the coPI/study coordinator - Dr. Bob Taylor: bob@tropmedres.ac
- the MORU pharmacovigilance officer

This initial contact can be a brief email or fax (+66 2 354 9169) to alert the team of a SAE. Thereafter, a SAE form will be completed. Follow up SAE reports will be sent as needed.

The study coordinator will then alert the DMEC.

The research teams will be responsible for reporting SAEs to any local regulatory or ethical body, if this is required.

9 DATA MONITORING & ETHICS COMMITTEE

An independent DMEC will be established and consist of qualified volunteers with the necessary knowledge of clinical trials and G6PD deficiency. There will also be a statistician.

A DMEC charter will be written that will be reviewed and approved by the DMEC. The charter will outline the DMEC responsibilities, frequency of meetings, and, notably, details of safety monitoring and which data the DMEC wishes to see in real time. The DMEC will review all data in the strictest confidence. Data that the DMEC will review will include:

- Hb data in the first 50 RDT diagnosed G6PDd patients in real time
- Hb data in the first 100 children admitted in Uganda to review the admissions policy
- all SAEs irrespective of the causality to study drugs
- AEs every 6 months.

10 MANAGEMENT OF SEVERE / PROFOUND ANAEMIA

10.1 General principles

In malaria, the Hb tends to fall initially before recovering. Anaemic African children with high parasite counts are prone to developing profound and severe anaemia that may require rescue with a blood transfusion. Detailed guidelines will be written on anaemia management and will be included in prestudy training.

10.2 Indications for blood transfusion include:

Children often tolerate lower Hb concentrations than adults, so their Hb thresholds for triggering a blood transfusion are generally lower.

The indications for transfusion are:

- Hb < 4 g/dL , irrespective of the clinical condition of the patient
- Hb < 5g/dL, if any of the following clinical features are present:
    - respiratory distress encompassing:
    - dyspnoea at rest
    - tachypnoea and
    - deep respirations
    - dyspnoea on mild exertion
    - acidosis (usually manifests as respiratory distress).
    - impaired consciousness
    - prostration

11 STATISTICS

11.1 Sample size

The sample size is based on demonstrating non-inferiority of ACT + SLDPQ compared to ACT alone with respect to the development of profound/severe anaemia in the G6PDd group. The rate of profound/severe anaemia in AL treated uncomplicated *P. falciparum* in Kinshasa is 1.5% [42].

For a non-inferiority margin of 2.5%, a two sided alpha=0.05 and a power of 80%, the sample size per group is 374 patients. Allowing for a 5% loss to follow up this become 393. We will recruit 800 G6PDd patients, the majority of whom will be males.

We will also recruit 800 patients into the G6PD normal arm, for a total sample size of 1600 patients. In the G6PD normal group, we expect the male-female ratio to be close to 1 and the age distribution to be very similar to that of the G6PDd group. The majority of malaria patients will be < 5 years old.

We plan to recruit a total of 1600 patients across both sites. Local ethical approval will be sought to recruit up to 1200 patients per site in case one of the sites experiences difficulties in recruitment.

Although genetic studies have shown relative high G6PDd allele frequencies, we are mindful of the weak correlation between G6PD genotypes and enzyme activities. Therefore, we have assumed a 10% proportion of G6PD RDT diagnosed G6PDd patients (most will be male) to calculate the number of patients we will need to screen ~8500 patients to obtain 800 G6PDd patients.

For other IBDs, we expect to recruit ~596 thalassaemia heterozygotes (prevalence 37.5%), ~246 sickle cell trait (15.7%), ~103 thalassaemia homozygotes (6.6%), and 14 sickle cell disease patients (0.9%).

11.2 Analyses & analysis populations

All patients who received at least one dose of ACT and PQ/placebo will be included in the analysis. All analyses will be detailed later in a prospectively written analysis plan. Below is a summary.

The main strategy for analysis of the primary end point will be assessed by chi squared or Fisher's exact test, as appropriate, calculating the 95% CIs of the risk difference to see if ACT + SLDPQ has achieved non-inferiority in the G6PDd group. Logistic regression will be used to adjust for potential confounders. Non inferiority will be assessed on the whole data set i.e. combining data from both sites using the lower end of the 95% confidence interval. Other proportional data will also be analysed by chi squared or Fisher's exact test.

Continuous data (e.g. Hb, biochemistry, parasite counts) between two groups will be analysed by the unpaired '*t*' test, transforming the data, as needed.

All analyses will be two sided and a p value of < 0.05 is considered statistically significant.

We will examine factors that are associated with: (i) the development of profound / severe anaemia (logistic regression), (ii) parameters of Hb recovery (multiple regression). *A priori*, we will explore the relationships between Hb profiles in the following: (i) G6PD status (defined by genotype and by enzyme activity), (ii) any IBD vs. not, (iii) age <5 vs. age ≥5y, (iv) high biomass infections, assessed by microscopy and measured HRP2 concentrations, and (v) males vs. females. For these analyses, estimates and 95% confidence intervals (CIs) will be presented but not p values.

The PK data for PQ and cPQ will be analysed by non-compartmental analysis. The following PK parameters will be estimated:

- Cmax
- Tmax
- Clearance
- Half life
- AUC
- Vd

For lumefantrine and piperaquine, drug absorption kinetics will be modelled over the first 24 hours.

The Day 7 concentrations of lumefantrine and piperaquine will be compared between PQ and placebo recipients. Depending on the lumefantrine and piperaquine data obtained, other modelled PK parameters will be determined, as the data permit.

12 ETHICS

12.1 Informed Consent

All caregivers/legal guardians of all patients must give informed consent before they can enter the study. They will be given a patient information sheet (PIS) to read and a member of the research team will give a verbal explanation.

The PIS will detail the nature of the study, follow up schedule, the known side effects of AL, DHAPP and PQ and any other risks involved in taking part in the study and the total blood volume. It will be clearly stated that the caregivers'/legal guardian's decision to allow her/his child to join this study is voluntary and if she/he declines, this will not have any adverse consequences for future treatment at the hospital or clinic.

Caregivers/legal guardians will be allowed as much time as they need to consider the information and will have the opportunity to question the site Principal Investigator/research team doctors or other independent parties to decide whether they will participate in the study.

Written Informed Consent will then be obtained by means of subject dated signature, and the signature of the research physician who obtained the consent. If the caregiver/legal guardian cannot read or write, a thumb print is acceptable. In this scenario, an independent witness must sign the consent form as well. A copy of the signed Informed Consent will be given to the subject. The original signed form will be retained at the study site.

In Uganda at Mbale, the PIS and consent form will be in English and several local languages:

- Luganda
- Lugwere
- Ateso
- Swahili
- Lumasaba

If another site is used e.g. at Soroti, the Ateso and Kumam languages will be used. In Kinshasa, the PIS will be in French and Lingala.

The PIS/ICFs for Uganda and Kinshasa have been prepared in accordance with the specific requirements of the local ethics committees and are version controlled for each country.

12.2 Approval by the national ethics committees

Before study initiation, official approval to conduct the study will be obtained from the relevant authorities to conduct the study and import the PQ/placebo.

In Uganda, these are:

- the Mbale Research Ethics Committee
- the National Drug Authority (NDA) and
- Uganda National Council of Science and Technology (UNCST)

In DRC, these are:

- the Kinshasa School of Public Heath EC and the Ministry of Health of DRC
- the drug regulatory authority

The protocol will also be submitted to the Oxford University Tropical Ethics Committee (OxTREC).

12.3 Risks

This is a low to moderate risk study. The drugs being used have long track records, their side effects are well known and there will be a good safety net in place to detect early patients with declining Hb concentrations who may need a blood transfusion.

12.3.1 Artemether lumefantrine

AL has been in use since 2001. It is registered in many countries, including the USA, and is used widely in Africa.

It is highly efficacious against uncomplicated *P. falciparum*, achieving cure rates of >92% [42] [57]. It is very well tolerated. Rates of early drug induced vomiting leading to AL discontinuation are < 1.6% in children (CoArtem® SPC). Moreover, there was no evidence of auditory nerve toxicity following repeated AL treatments [58].

12.3.2 Dihydroartemisinin piperaquine

DHAPP has been used extensively in China and more recently in Africa and Asia. It has an excellent safety record and is very well tolerated. Side effects are reported with quite marked ranges in different studies [45] and include:

- early vomiting which is higher in younger children aged < 5y. Rates of 18 to 3% have been reported in children aged <1y and aged 4-5y, respectively.
- vomiting leading to a change in treatment is also dependent on age and was 3.8% in Ugandan children aged 6 to 24 months [41]
- anorexia 11%
- nausea, diarrhoea, abdominal pain 3-10%
- headache 23%
- dizziness 1.5-17%
- poor sleep 14%
- itching 1-2%
- urticaria 1%

12.3.3 Primaquine

PQ is also generally well tolerated. The main side effects are abdominal pain which is dose related and improved by taking PQ with food [28]. Rates of abdominal pain and/or cramps were 3% (15 mg/d), 12.5% (22.5 mg/d), 10% (30 mg/d) and 5/7 (60 mg/d).

PQ converts haemoglobin (Hb) to methaemoglobin (metHb). Blue discolouration around the lips may be seen when metHb concentrations are between 15-20 g/L, equivalent to ~10-20% of the Hb concentration. It is rare - only one case was found in 3,000 American soldiers on 15 mg of daily for 14 days [59].

MetHb levels exceeding 10% with PQ doses of 15 or 22.5 mg/day in adults are unusual; levels of 16-18% were found in three (6.5%) of 46 Australian soldiers. [52] In vivax infected Cambodians, the maximum metHb after one dose of DHAPP and one dose of 45 mg of PQ was 4.9% [54] with a significantly (p<0.001) lower median value in the G6PDd (0.7%) vs. the G6PD normal (1.7%) patients (Bob Taylor, unpublished).

There are reports of both increases and decreases in the total white cell counts but the clinical significance of these findings is unclear; some patients also received myelotoxic drugs or had diseases characterised by myelosuppression are additional risk factors [28].

The most important side effect of PQ is AHA in individuals with G6PD deficiency. Its severity is related to the degree of G6PD deficiency and the PQ dose. Haemolysis is less severe in the African (A^-) G6PD variant compared to the Asian and Mediterranean variants [60].

In *P. falciparum* infected Tanzanian children [all with haemoglobin (Hb) concentrations > 8g/dL], 0.75 mg/kg of PQ caused a fall in the mean Hb concentration in all patients that was higher in G6PDd patients: -2.5 (G6PD A- hemizygous or homozygous) vs. -1.6 (G6PD A- heterozygotes) vs. -0.5 g/dL (G6PD B / wild type homozygous). In this study, the target PQ dose was 0.75 mg/kg but many patients received a PQ dose exceeding this.

Our study will be using SLDPQ; the target dose 0.25 mg/kg and maximum dose will be 0.45 mg/kg in children aged 1 year. Therefore, we expect smaller falls in Hb compared to the Tanzanian study.

Very limited data in falciparum infected patients in Cambodia with the severe G6PD variant, Viangchan, suggest that SLDPQ, dosed exactly at 0.25 mg/kg, had little additional haemolytic effect compared to no SLDPQ (Bob Taylor, unpublished observations). Similarly, Bancone *et al* showed that in villages on the Thai Burmese border, G6PDd and G6PD normal, healthy individuals had small declines in mean Hb of approximately 5 and 1 %, respectively, when given DHAPP plus SLDPQ, also dosed exactly at 0.25 mg base/kg [61].

12.3.4 Blood sampling

Blood taking causes discomfort but this tends to be transient. Rarely, a venepuncture site may become infected. For the PK sampling on D0, an IV catheter will remain in place for 24h. Full aseptic techniques will be used when taking blood to minimise the infection risk.

12.4 Benefits

Patients who enrol into this study will benefit from the close follow-up by the study doctors of their malaria episode and any other minor illnesses that occur during the follow-up period.

In Kinshasa, they will also be benefit from knowing their G6PD and sickle cell (HbS) status for which they will receive adequate information and counselling.

The analysis of thalassaemia will take place after study completion and if any patient has a major thalassaemia that was previously unknown, we will attempt to trace that patient and inform his/her caregivers/guardians of the result. .

Adding PQ to ACT treatment of malaria and the information obtained will benefit the wider community because if scaled up, SLDPQ should result in a further reduction in malaria transmission

12.5 Alternatives to joining the study

If patients or guardians do not allow their child to participate in this study or their child does not meet the entry criteria, they will be treated in accordance with the local guidelines.

12.6 Patient reimbursements

In Kinshasa the patient will be fully taken in charge by the study for the period of admission in the health centre and money will provided to pay the transport for the follow-up visits. No other payments will be made.

In Uganda, inpatient care will be free and reasonable transport costs will also be reimbursed for caregivers attending follow-up.

12.7 Confidentiality

All information on patients will remain confidential and be shared only by the study team. Unique identifiers will be used for computer-based data entry and blood samples. Patient names will not appear on any study related documents except the enrolment log.

The site principal investigator will ensure that all study related documents and screening are kept in locked files and that access to them is limited.

12.8 Clinical trials insurance

The University of Oxford has a specialist insurance policy in place from Newline Underwriting Management Ltd, at Lloyd’s of London. This would operate in the event of any participant suffering harm as a result of their involvement in the research.

12.9 Clinical trial registration

This trial will be registered with the ISRCTN registry.

12.10 Monitoring procedures

The study sites will be visited by the Monitor periodically at times agreed on with the site PIs. It is the function of the Monitor to ascertain if all aspects of the study protocol are complied with and that the conduct of the study conforms to applicable regulatory requirements and established rules for GCP.

At the time of each monitoring visit, the Monitor will review the completed CRFs to ascertain that all items have been completed and that the data provided are accurate and obtained in the manner specified in the protocol.

The Monitor will also check that the data in the CRF are consistent with the clinical records (Source Data Verification [SDV]) and that study results are recorded completely and correctly. The frequency of monitoring visits and the extent of SDV will be specified in the monitoring plan. The Monitor will check on the reporting of SAEs and the procedures for IMP accountability and record keeping. For this purpose the Monitor must be given direct access to clinical records, original laboratory data, etc., as far as these relate to the study and without jeopardizing Subject integrity. CRFs for all included Subjects must be made available to the Monitor for review.

Data and all appropriate documentation will be stored after the completion of the study, including the follow-up period, for the minimum length of time according to local requirements.

13 DATA HANDLING AND RECORD KEEPING

All study data will be recorded onto a paper case record form (CRF). Data derived from source documents should be consistent with the source documents, or the discrepancies should be explained. Any change or correction to a case report form should be dated and explained and should not obscure the original entry. All case report forms will be checked for completeness.

If sites are unable to enter data locally, the CRFs will be sent to MORU for data entry using Macro, cleaned and analysed using STATA. The subject will be identified by a study specific subject number and/or code. The name and any other identifying detail will NOT be included in any study data electronic file.

The site principal investigator is responsible for ensuring data collection and entry are accurate and complete and for keeping all screening forms, the case report form and the completed subject identification code list in a secure location.

The data will be analysed at MORU by the study statistician.

14 FINANCING

This study is being funded by the UK Medical Research Council, the Wellcome Trust of Great Britain and UK Aid.

15 DATA OWNERSHIP & PUBLICATION POLICY

The data generated in this study belongs to the study group as a whole. The final database will be shared amongst the site PIs and key members of the research team.

The database may be shared with investigators not directly involved in this study but only after the main paper has been published. There will be a data sharing committee. The database will only be shared if future publications are not compromised.

The criteria for authorship will be consistent with the international guidelines (http://www.icmje.org/#author).

16 REFERENCESS

1. White NJ, Qiao LG, Qi G, Luzzatto L. Rationale for recommending a lower dose of primaquine as a Plasmodium falciparum gametocytocide in populations where G6PD deficiency is common. Malar J 2012;11:418.

2. Committee WHOMPA, Secretariat. Malaria Policy Advisory Committee to the WHO: conclusions and recommendations of September 2012 meeting. Malar J 2012;11:424.

3. Noedl H, Se Y, Schaecher K, et al. Evidence of artemisinin-resistant malaria in western Cambodia. The New England journal of medicine 2008;359:2619-20.

4. Dondorp AM, Nosten F, Yi P, et al. Artemisinin resistance in Plasmodium falciparum malaria. N Engl J Med 2009;361:455-67.

5. Ariey F, Witkowski B, Amaratunga C, et al. A molecular marker of artemisinin-resistant Plasmodium falciparum malaria. Nature 2014;505:50-5.

6. Phyo AP, Nkhoma S, Stepniewska K, et al. Emergence of artemisinin-resistant malaria on the western border of Thailand: a longitudinal study. Lancet 2012;379:1960-6.

7. Hien TT, Thuy-Nhien NT, Phu NH, et al. In vivo susceptibility of Plasmodium falciparum to artesunate in Binh Phuoc Province, Vietnam. Malaria journal 2012;11:355.

8. Ashley EA, Dhorda M, Fairhurst RM, et al. Spread of artemisinin resistance in Plasmodium falciparum malaria. The New England journal of medicine 2014;371:411-23.

9. Amaratunga C, Sreng S, Suon S, et al. Artemisinin-resistant Plasmodium falciparum in Pursat province, western Cambodia: a parasite clearance rate study. The Lancet infectious diseases 2012;12:851-8.

10. Leang R, Barrette A, Bouth DM, et al. Efficacy of dihydroartemisinin-piperaquine for treatment of uncomplicated Plasmodium falciparum and Plasmodium vivax in Cambodia, 2008 to 2010. Antimicrobial agents and chemotherapy 2013;57:818-26.

11. Leang R, Taylor WR, Bouth DM, et al. Evidence of Plasmodium falciparum Malaria Multidrug Resistance to Artemisinin and Piperaquine in Western Cambodia: Dihydroartemisinin-Piperaquine Open-Label Multicenter Clinical Assessment. Antimicrobial agents and chemotherapy 2015;59:4719-26.

12. Spring MD, Lin JT, Manning JE, et al. Dihydroartemisinin-piperaquine failure associated with a triple mutant including kelch13 C580Y in Cambodia: an observational cohort study. The Lancet infectious diseases 2015.

13. Amaratunga C, Lim P, Suon S, et al. Dihydroartemisinin-piperaquine resistance in Plasmodium falciparum malaria in Cambodia: a multisite prospective cohort study. The Lancet infectious diseases 2016;16:357-65.

14. Tun KM, Imwong M, Lwin KM, et al. Spread of artemisinin-resistant Plasmodium falciparum in Myanmar: a cross-sectional survey of the K13 molecular marker. The Lancet infectious diseases 2015;15:415-21.

15. Dicko A, Brown JM, Diawara H, et al. Primaquine to reduce transmission of Plasmodium falciparum malaria in Mali: a single-blind, dose-ranging, adaptive randomised phase 2 trial. The Lancet infectious diseases 2016;16:674-84.

16. Eziefula AC, Bousema T, Yeung S, et al. Single dose primaquine for clearance of Plasmodium falciparum gametocytes in children with uncomplicated malaria in Uganda: a randomised, controlled, double-blind, dose-ranging trial. The Lancet infectious diseases 2014;14:130-9.

17. Shekalaghe SA, ter Braak R, Daou M, et al. In Tanzania, hemolysis after a single dose of primaquine coadministered with an artemisinin is not restricted to glucose-6-phosphate dehydrogenase-deficient (G6PD A-) individuals. Antimicrobial agents and chemotherapy 2010;54:1762-8.

18. Moore BR, Salman S, Benjamin J, et al. Pharmacokinetic properties of single-dose primaquine in Papua New Guinean children: feasibility of abbreviated high-dose regimens for radical cure of vivax malaria. Antimicrobial agents and chemotherapy 2014;58:432-9.

19. Barnes KI, Little F, Smith PJ, Evans A, Watkins WM, White NJ. Sulfadoxine-pyrimethamine pharmacokinetics in malaria: pediatric dosing implications. Clin Pharmacol Ther 2006;80:582-96.

20. Tarning J, Chotsiri P, Jullien V, et al. Population pharmacokinetic and pharmacodynamic modeling of amodiaquine and desethylamodiaquine in women with Plasmodium vivax malaria during and after pregnancy. Antimicrob Agents Chemother 2012;56:5764-73.

21. Pybus BS, Sousa JC, Jin X, et al. CYP450 phenotyping and accurate mass identification of metabolites of the 8-aminoquinoline, anti-malarial drug primaquine. Malar J 2012;11:259.

22. Constantino L, Paixao P, Moreira R, Portela MJ, Do Rosario VE, Iley J. Metabolism of primaquine by liver homogenate fractions. Evidence for monoamine oxidase and cytochrome P450 involvement in the oxidative deamination of primaquine to carboxyprimaquine. Exp Toxicol Pathol 1999;51:299-303.

23. Thompson SF, Fraser IM, Strother A, Bull BS. Change of deformability and Heinz body formation in G6PD-deficient erythrocytes treated with 5-hydroxy-6-desmethylprimaquine. Blood Cells 1989;15:443-52; discussion 53-4.

24. Bowman ZS, Jollow DJ, McMillan DC. Primaquine-induced hemolytic anemia: role of splenic macrophages in the fate of 5-hydroxyprimaquine-treated rat erythrocytes. J Pharmacol Exp Ther 2005;315:980-6.

25. Ganesan S, Chaurasiya ND, Sahu R, Walker LA, Tekwani BL. Understanding the mechanisms for metabolism-linked hemolytic toxicity of primaquine against glucose 6-phosphate dehydrogenase deficient human erythrocytes: evaluation of eryptotic pathway. Toxicology 2012;294:54-60.

26. Bolchoz LJ, Budinsky RA, McMillan DC, Jollow DJ. Primaquine-induced hemolytic anemia: formation and hemotoxicity of the arylhydroxylamine metabolite 6-methoxy-8-hydroxylaminoquinoline. J Pharmacol Exp Ther 2001;297:509-15.

27. Bolchoz LJ, Morrow JD, Jollow DJ, McMillan DC. Primaquine-induced hemolytic anemia: effect of 6-methoxy-8-hydroxylaminoquinoline on rat erythrocyte sulfhydryl status, membrane lipids, cytoskeletal proteins, and morphology. J Pharmacol Exp Ther 2002;303:141-8.

28. Clayman CB, Arnold J, Hockwald RS, Yount EH, Jr., Edgcomb JH, Alving AS. Toxicity of primaquine in Caucasians. J Am Med Assoc 1952;149:1563-8.

29. Liu H, Tekwani BL, Nanayakkara NP, Walker LA, Doerksen RJ. Methemoglobin generation by 8-aminoquinolines: effect of substitution at 5-position of primaquine. Chem Res Toxicol 2013;26:1801-9.

30. Allahyari R, Strother A, Fraser IM, Verbiscar AJ. Synthesis of certain hydroxy analogues of the antimalarial drug primaquine and their in vitro methemoglobin-producing and glutathione-depleting activity in human erythrocytes. J Med Chem 1984;27:407-10.

31. Bennett JW, Pybus BS, Yadava A, et al. Primaquine failure and cytochrome P-450 2D6 in Plasmodium vivax malaria. The New England journal of medicine 2013;369:1381-2.

32. Ingelman-Sundberg M. Genetic polymorphisms of cytochrome P450 2D6 (CYP2D6): clinical consequences, evolutionary aspects and functional diversity. The pharmacogenomics journal 2005;5:6-13.

33. Crews KR, Gaedigk A, Dunnenberger HM, et al. Clinical Pharmacogenetics Implementation Consortium guidelines for cytochrome P450 2D6 genotype and codeine therapy: 2014 update. Clin Pharmacol Ther 2014;95:376-82.

34. Owen RP, Sangkuhl K, Klein TE, Altman RB. Cytochrome P450 2D6. Pharmacogenetics and genomics 2009;19:559-62.

35. Chen I, Poirot E, Newman M, et al. An assessment of the supply, programmatic use, and regulatory issues of single low-dose primaquine as a Plasmodium falciparum gametocytocide for sub-Saharan Africa. Malar J 2015;14:204.

36. Kim S, Nguon C, Guillard B, et al. Performance of the CareStart G6PD deficiency screening test, a point-of-care diagnostic for primaquine therapy screening. PloS one 2011;6:e28357.

37. Espino FE, Bibit JA, Sornillo JB, Tan A, von Seidlein L, Ley B. Comparison of Three Screening Test Kits for G6PD Enzyme Deficiency: Implications for Its Use in the Radical Cure of Vivax Malaria in Remote and Resource-Poor Areas in the Philippines. PloS one 2016;11:e0148172.

38. Roca-Feltrer A, Khim N, Kim S, et al. Field trial evaluation of the performances of point-of-care tests for screening G6PD deficiency in Cambodia. PloS one 2014;9:e116143.

39. Johnson MK, Clark TD, Njama-Meya D, Rosenthal PJ, Parikh S. Impact of the method of G6PD deficiency assessment on genetic association studies of malaria susceptibility. PloS one 2009;4:e7246.

40. Shah SS, Macharia A, Makale J, et al. Genetic determinants of glucose-6-phosphate dehydrogenase activity in Kenya. BMC Med Genet 2014;15:93.

41. Creek D, Bigira V, Arinaitwe E, et al. Increased risk of early vomiting among infants and young children treated with dihydroartemisinin-piperaquine compared with artemether-lumefantrine for uncomplicated malaria. The American journal of tropical medicine and hygiene 2010;83:873-5.

42. Onyamboko AM, Fanello CI, Wongsaen K, et al. A randomized comparison of the efficacy and tolerability of three artemisinin-based combination treatments for children with acute falciparum malaria in The Democratic Republic of Congo. Antimicrobial agents and chemotherapy 2014.

43. Adjei A, Narh-Bana S, Amu A, et al. Treatment outcomes in a safety observational study of dihydroartemisinin/piperaquine (Eurartesim((R))) in the treatment of uncomplicated malaria at public health facilities in four African countries. Malar J 2016;15:43.

44. Baiden R, Oduro A, Halidou T, et al. Prospective observational study to evaluate the clinical safety of the fixed-dose artemisinin-based combination Eurartesim(R) (dihydroartemisinin/piperaquine), in public health facilities in Burkina Faso, Mozambique, Ghana, and Tanzania. Malar J 2015;14:160.

45. Zwang J, Ashley EA, Karema C, et al. Safety and efficacy of dihydroartemisinin-piperaquine in falciparum malaria: a prospective multi-centre individual patient data analysis. PloS one 2009;4:e6358.

46. Lynch T, Price A. The effect of cytochrome P450 metabolism on drug response, interactions, and adverse effects. Am Fam Physician 2007;76:391-6.

47. Schneider P, Reece SE, van Schaijk BC, et al. Quantification of female and male Plasmodium falciparum gametocytes by reverse transcriptase quantitative PCR. Mol Biochem Parasitol 2015;199:29-33.

48. Medina Lara A, Mundy C, Kandulu J, Chisuwo L, Bates I. Evaluation and costs of different haemoglobin methods for use in district hospitals in Malawi. J Clin Pathol 2005;58:56-60.

49. Malaria Genomic Epidemiology N, Malaria Genomic Epidemiology N. Reappraisal of known malaria resistance loci in a large multicenter study. Nat Genet 2014;46:1197-204.

50. Waterfall CM, Cobb BD. Single tube genotyping of sickle cell anaemia using PCR-based SNP analysis. Nucleic acids research 2001;29:E119.

51. Williams TN, Wambua S, Uyoga S, et al. Both heterozygous and homozygous alpha+ thalassemias protect against severe and fatal Plasmodium falciparum malaria on the coast of Kenya. Blood 2005;106:368-71.

52. Sietsma A, Naughton MA, Harley JD. Methaemoglobin levels in soldiers receiving antimalarial drugs. Med J Aust 1971;1:473-5.

53. Fryauff DJ, Baird JK, Basri H, et al. Randomised placebo-controlled trial of primaquine for prophylaxis of falciparum and vivax malaria. Lancet 1995;346:1190-3.

54. Kheng S, Muth S, Taylor WR, et al. Tolerability and safety of weekly primaquine against relapse of Plasmodium vivax in Cambodians with glucose-6-phosphate dehydrogenase deficiency. BMC Med 2015;13:203.

55. White NJ, van Vugt M, Ezzet F. Clinical pharmacokinetics and pharmacodynamics and pharmacodynamics of artemether-lumefantrine. Clin Pharmacokinet 1999;37:105-25.

56. Creek DJ, Bigira V, McCormack S, et al. Pharmacokinetic predictors for recurrent malaria after dihydroartemisinin-piperaquine treatment of uncomplicated malaria in Ugandan infants. The Journal of infectious diseases 2013;207:1646-54.

57. Hamed K, Grueninger H. Coartem((R)): a decade of patient-centric malaria management. Expert Rev Anti Infect Ther 2012;10:645-59.

58. Carrasquilla G, Baron C, Monsell EM, et al. Randomized, prospective, three-arm study to confirm the auditory safety and efficacy of artemether-lumefantrine in Colombian patients with uncomplicated Plasmodium falciparum malaria. The American journal of tropical medicine and hygiene 2012;86:75-83.

59. Hansen JE, Cleve EA, Pruitt FW. Relapse of vivax malaria treated with primaquine and report of one case of cyanosis (methemoglobinemia) due to primaquine. Am J Med Sci 1954;227:9-12.

60. Beutler E, Duparc S. Glucose-6-phosphate dehydrogenase deficiency and antimalarial drug development. The American journal of tropical medicine and hygiene 2007;77:779-89.

61. Bancone G, Chowwiwat N, Somsakchaicharoen R, et al. Single Low Dose Primaquine (0.25mg/kg) Does Not Cause Clinically Significant Haemolysis in G6PD Deficient Subjects. PloS one 2016;11:e0151898.

17 APPENDIX 1: STUDY SCHEDULE – KINSHASA & UGANDA INPATIENTS

	D0								D1		D2		D3		D4	D5	D6	D7	D14	D21	D28	D35	D42	Drec	Blood volumes	total
	H0	H1	H1.5	H2	H4	H6	H8	H12	H24	H36	H48	H60	H72	H84	H96											
Initial patient management																										
Verbal consent (routine in DRC)	X																									
Brief history & examination	X																									
Hb (HemoCue)	X																								0.02	0.02
Malaria RDT	X																								0.02	0.02
Malaria blood film (Kinshasa only if malaria RDT positive)	X																								0.05	0.05
G6PD RDT qualitative	X																								0.02	0.02
Enrolled patients																										
Written consent																										
Assign study number	X																									
Open question about well being	X								X		X		X		X	X	X	X	X	X	X	X	X	X		
Symptoms checklist	X												X					X	X	X	X	X	X	X		
Weight	X																									
Vital signs	X							X	X	X	X	X	X	X	X	X	X	X	X	X	X	X	X	X		
General examination†	X																									
MetHb oximeter	X					X		X	X	X	X	X	X	X	X	X	X	X	X	X	X	X	X			
Drug administration																										
AL	X								X		X															
DHAPP	X								X		X															
Primaquine/placebo	X																									
Laboratory tests - finger sticks																										
Malaria blood films	X					X		X	X	X	X	X	X	X	X	X	X	X	X	X	X	X	X	X	0.05	1
Hb (HemoCue)						X		X	X	X	X	X	X	X	X	X	X	X	X	X	X	X	X	X	0.05	1
Haematocrit - microcentrifuge	X					X		X	X	X	X	X	X	X	X	X	X	X	X	X	X	X	X	X	0.05	1
Thin film for retic count	X												X					X	X	X	X	X	X		0.05	0.45
G6PD Biosensor	X																								0.03	0.03
Hb colour card	X												X					X			X				0.03	0.12
Gametocyte qPCR	X												X					X	X	X	X	X	X		0.1	0.08
Filter paper samples																										
Filter paper blood spot - malaria genotype	X																	X	X	X	X	X	X	X	0.05	0.04
Filter paper molecular markers of resistance	X																								0.05	0.05
Venous sample																										
FBC EDTA#	X												X					X			X				2	8
Biochemistry-heparin‡	X												X					X			X				2	8
Lumefantrine/piperaquine (EDTA)	X												X					X			X				2	8
Primaquine*	X	X	X	X	X	X	X	X	X																0.75	6
																										**33.8**

D0 is the first day of dosing for all patients
Do investigations on D4-6 only if patient is still admitted for medical reasons
† Vital signs includes colour of lips & conjunctivae.
Do physical examination as clinically indicated post D0
Drec = day of recurrence
* Primaquine - in a subset
# EDTA: G6PD activity, PCR studies for G6PD, haemoglobinopathies & CYP2D6
‡ AST, ALT, LDH, total bilirubin, conjugated bilirubin, creatinine, (store for haptoglobin + folate)

18 APPENDIX 2: STUDY SCHEDULE – UGANDA OUTPATIENTS

																			Blood volumes	
Initial patient management																				**total**
Verbal consent	X																			
Brief history & examination	X																			
Hb (HemoCue)	X																		0.02	0.02
Malaria RDT	X																		0.02	0.02
G6PD RDT qualitative	X																		0.02	0.02
	**D0**								**D1**	**D2**	**D3**	**D7**	**D14**	**D21**	**D28**	**D35**	**D42**	**Drec**		
Enrolled patients	**H0**	**H1**	**H1.5**	**H2**	**H4**	**H6**	**H8**	**H12**	**H24**											
Written consent	X																			
Assign study number	X																			
Open question about well being	X								X		X	X	X	X	X	X	X	X		
Symptoms checklist	X								X			X	X	X	X	X	X	X		
Weight	X																			
Vital signs	X								X	X	X	X	X	X	X	X	X	X		
General examination†	X																			
MetHb oximeter	X								X	X	X	X	X	X	X	X	X			
Drug administration																				
AL	X								X	X										
DHAPP	X								X	X										
Primaquine 0.25 mg/kg	X																			
Laboratory tests																				
Malaria blood films	X								X	X	X	X	X	X	X	X	X	X	0.05	0.75
Hb (HemoCue)	X								X	X	X	X	X	X	X	X	X	X	0.05	0.75
G6PD Biosensor	X																		0.03	0.03
Gametocyte qPCR	X											X	X	X	X	X	X		0.1	0.7
Filter paper blood spot - malaria genotype	X										X	X	X	X	X	X	X	X	0.05	0.4
Filter paper - molec markers of resistance	X																			
Venous sample																				
FBC EDTA#	X										X	X			X				2	8
Biochemistry-heparin‡	X										X	X			X				2	8
Lumefantrine/piperaquine (EDTA)	X										X	X			X				2	8
Primaquine*	X	X	X	X	X	X	X	X	X										0.75	6
																				**33**

D0 is the first day of dosing for all patients
Drec = day of recurrence
† Vital signs includes colour of lips & conjunctivae.
Do physical examination as clinically indicated post D0
EDTA: G6PD activity, PCR studies for G6PD, haemoglobinopathies & CYP2D6
‡ AST, ALT, LDH, total bilirubin, conjugated bilirubin, creatinine, (store for haptoglobin + folate)

19 APPENDIX 3. WHO CLASSIFICATION OF THERAPEUTIC EFFICACY

Early treatment failure (ETF)

- Danger signs or severe malaria on day 1, 2 or 3, in the presence of parasitaemia;
- Parasitaemia on day 2 higher than on day 0, irrespective of axillary temperature;
- Parasitaemia on day 3 with axillary temperature ≥ 37.5 °C; and parasitaemia on day 3 ≥ 25% of count on day 0.

Late clinical failure (LCF)

Danger signs or severe malaria in the presence of parasitaemia on any day between day 4 and day 42 in patients who did not previously meet any of the criteria of early treatment failure; and axillary temperature ≥ 37.5 °C in the presence of parasitaemia on any day between day 4 and day 42 in patients who did not previously meet any of the criteria of early treatment failure.

Late parasitological failure (LPF)

Presence of parasitaemia between day 7 and day 42 with a temperature < 37.5 °C in patients who did not previously meet any of the criteria of early treatment failure or late clinical failure.

Adequate clinical and parasitological response (ACPR)

- Absence of parasitaemia on day 42, irrespective of axillary temperature, in patients who did not previously meet any of the criteria of early treatment failure, late clinical failure or late parasitological failure.

The number of cases of **Total Treatment Failure (TTF)** is computed as ETF+LCF+LPF. After day 14 only parasitaemia confirmed by PCR as recrudescence is considered a treatment failure.
