# Supplementary figures and images for: Pharmacokinetics of single low dose primaquine in Ugandan and Congolese children with falciparum malaria
Source: eBioMedicine. 2023 Sep 25;96:104805. doi: 10.1016/j.ebiom.2023.104805 (PMC10550634; doi:10.1016/j.ebiom.2023.104805)

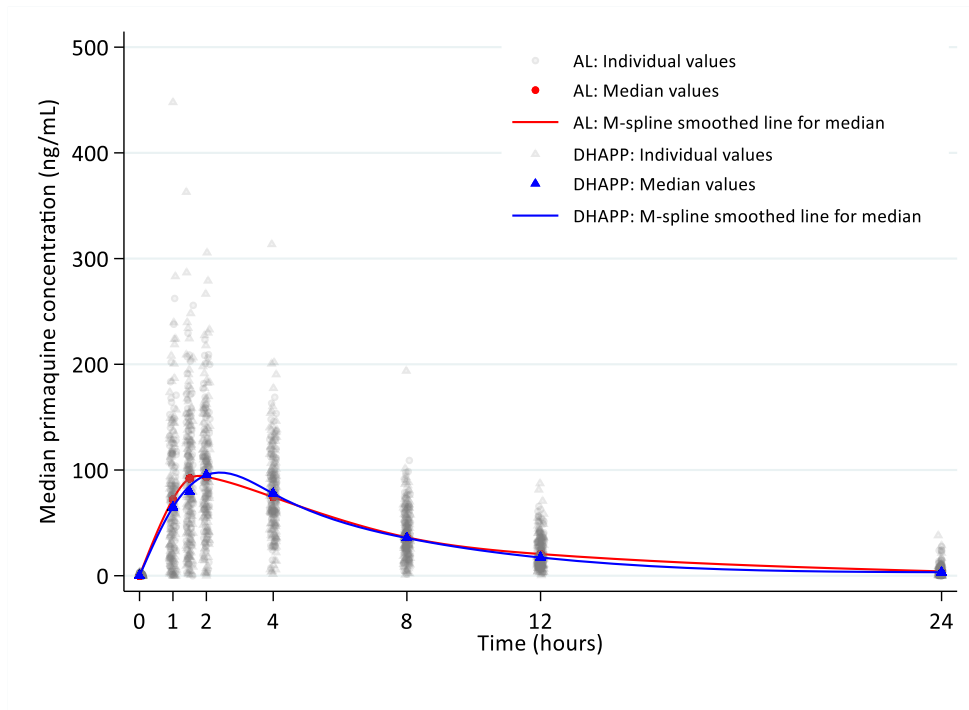

Supplement: Figure S1 [file mmc3.pdf]

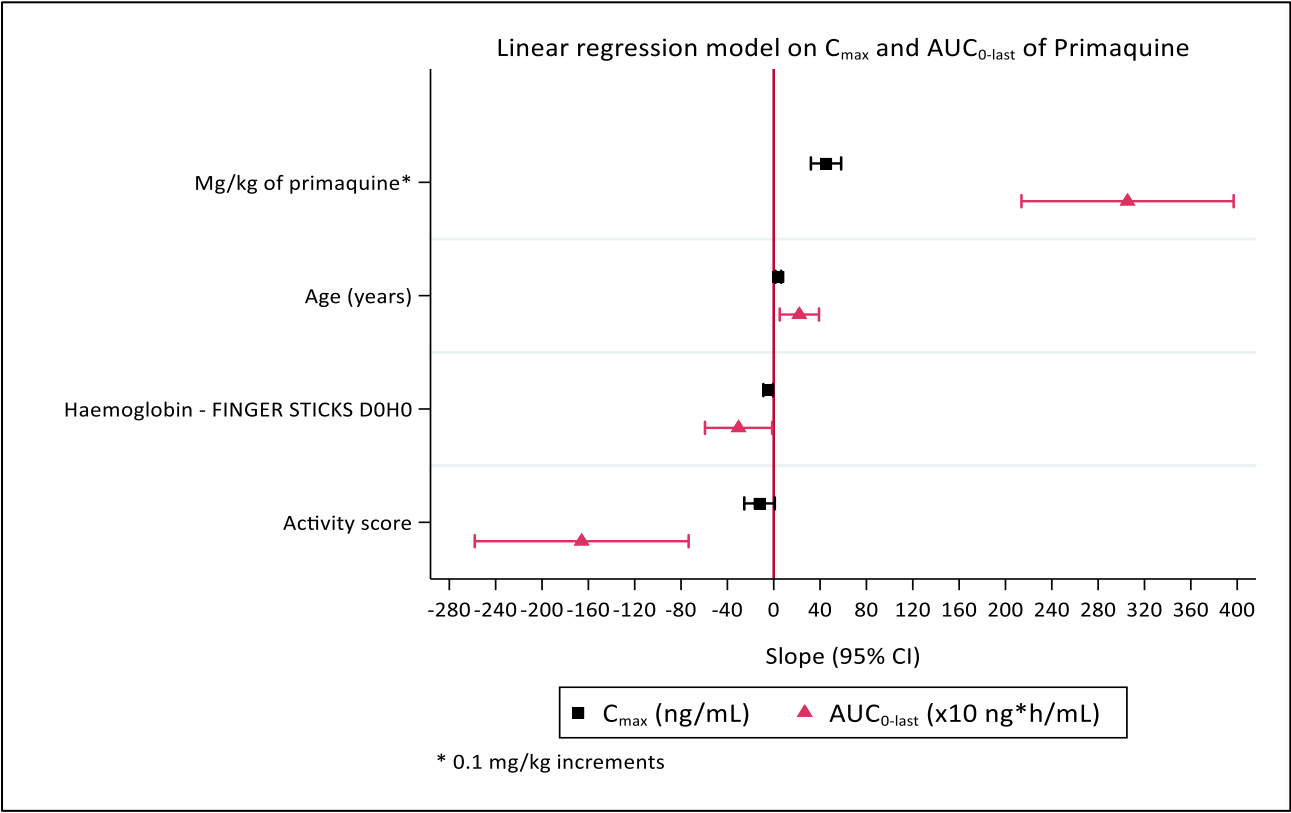

Supplement: Figure S2 [file mmc4.pdf]

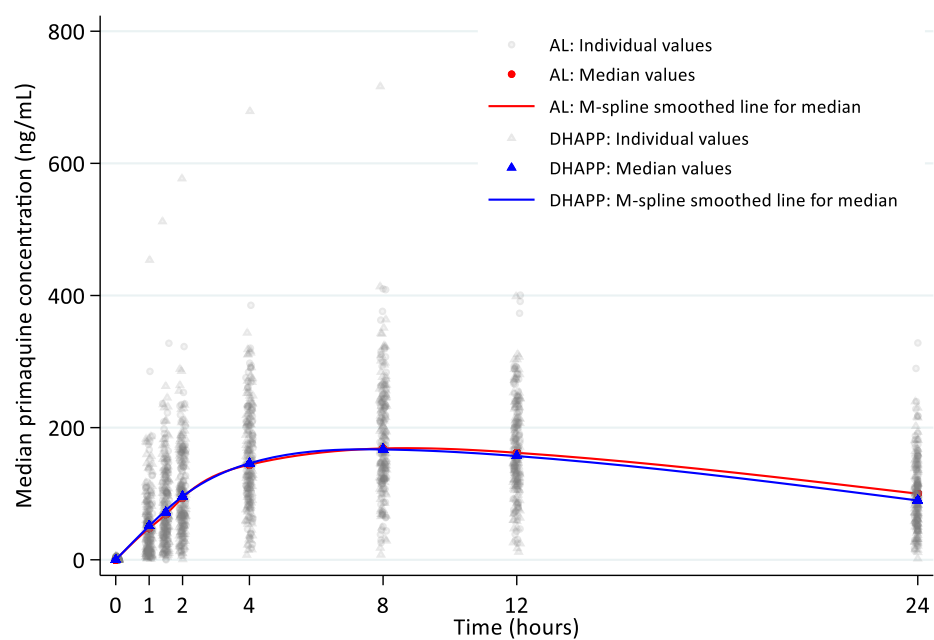

Supplement: Figure S5 [file mmc7.pdf]
