# Supplementary material for: Pharmacokinetics of single low dose primaquine in Ugandan and Congolese children with falciparum malaria
Source: eBioMedicine. 2023 Sep 25;96:104805. doi: 10.1016/j.ebiom.2023.104805 (PMC10550634; doi:10.1016/j.ebiom.2023.104805)
Supplement: Figure S3 [file mmc5.pdf]

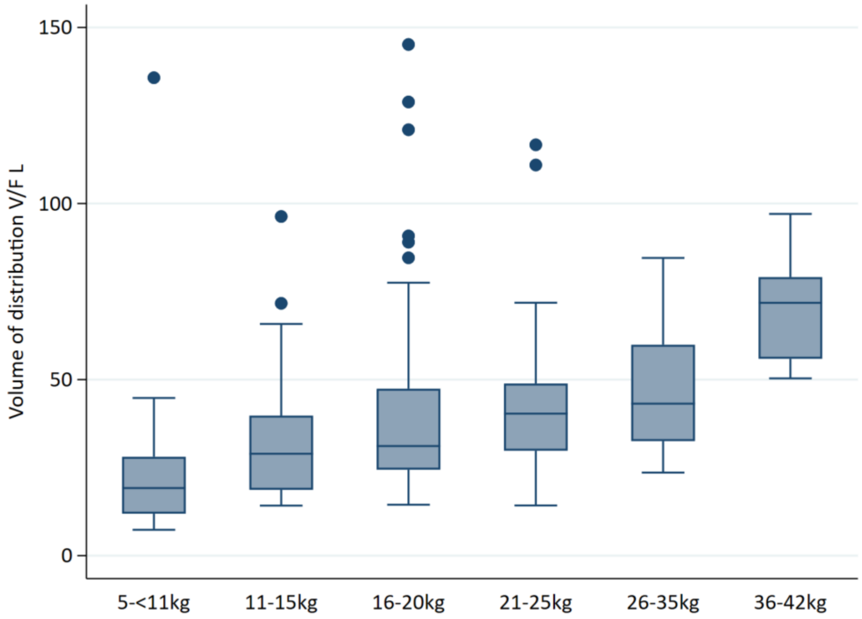

The medians (IQR) for 5-<11; 11-15kg; 16-20kg; 21-25kg; 26-35kg; and 36-42kg are 20.3 (11.9-28.5), 30.5 (20.1-46.1), 32.0 (25.1-48.3), 40.3 (30.2-49), 43.2 (32.5-59.9) and 71.8 (55.9-79.1), respectively.

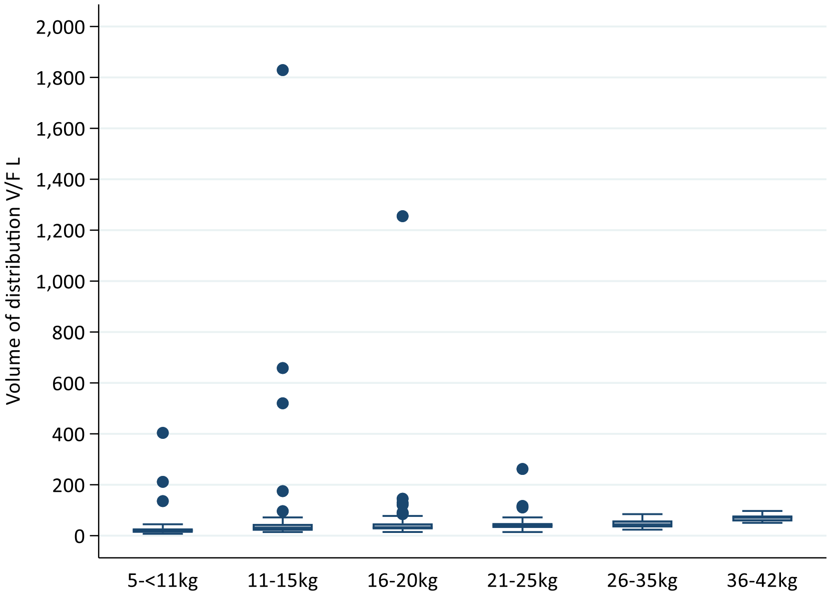

Outlying values have been removed for greater clarity: 174.9, 211.3, 261.9, 403.8, 520.0, 658.4, 1255.0, and 1828.7 L.
