# Supplementary material for: Pharmacokinetics of single low dose primaquine in Ugandan and Congolese children with falciparum malaria
Source: eBioMedicine. 2023 Sep 25;96:104805. doi: 10.1016/j.ebiom.2023.104805 (PMC10550634; doi:10.1016/j.ebiom.2023.104805)

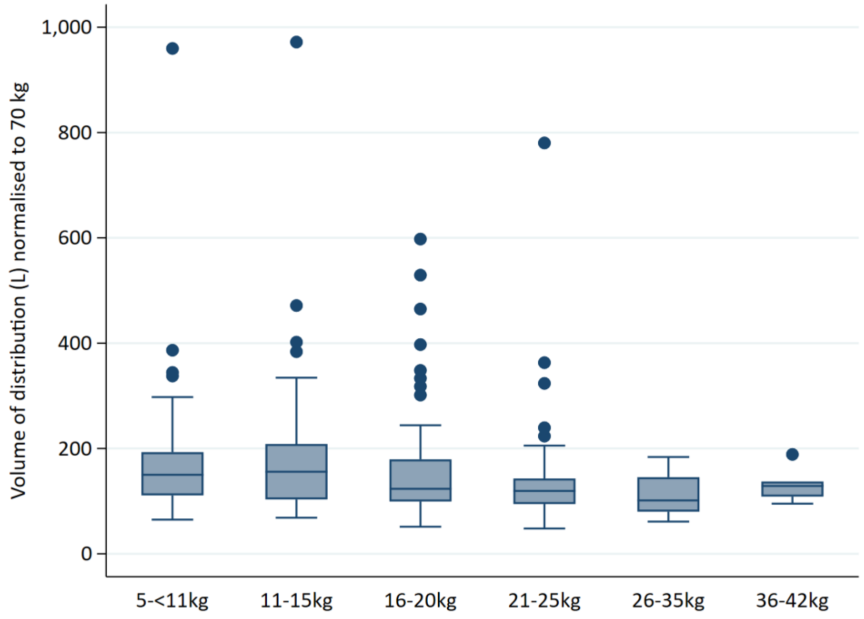

Six values > 1,000 L have been excluded for greater clarity: 1,479 & 2,826 (5-10 kg), 2,800, 3200 & 10,323 (11-15 kg), and 4,880 (16-20 kg) L. The medians (IQR) for 5-<11; 11-15kg; 16-20kg; 21-25kg; 26-35kg; and 36-42kg are 150.9 (111-244.3), 164.8 (107-229.8), 123.5 (101.2-180.2), 119.4 (94.5-142.9), 101.4 (80.1-145.4) and 128.9 (108.7-137.3), respectively.

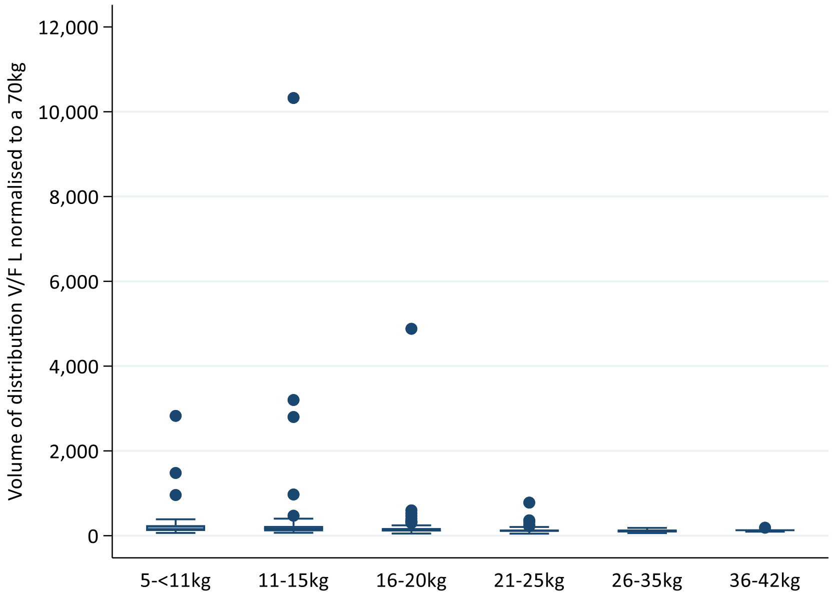

Supplement: Figure S4 [file mmc6.pdf]
